# Supplementary material for: Construction of Zn-doped RuO2 nanowires for efficient and stable water oxidation in acidic media
Source: Nat Commun. 2023 May 2;14:2517. doi: 10.1038/s41467-023-38213-1 (PMC10154325; doi:10.1038/s41467-023-38213-1)
Supplement: Supplementary file 1 — Supplementary Information [file 41467_2023_38213_MOESM1_ESM.pdf]

Supplementary Information

Construction of Zn-doped RuO_2 nanowires for efficient and stable water oxidation in acidic media

Dafeng Zhang[1,5], Mengnan Li[1,5], Xue Yong[2,3,5], Haoqiang Song[2], Geoffrey I.N. Waterhouse[4], Yunfei Yi[1], Bingjie Xue[1], Dongliang Zhang[1], Baozhong Liu[1,*], Siyu Lu[2,*]

[1]State Collaborative Innovation Center of Coal Work Safety and Clean-efficiency Utilization, Henan Key Laboratory of Coal Green Conversion, College of Chemistry and Chemical Engineering, Henan Polytechnic University, Jiaozuo 454003, P. R. China.

[2]Green Catalysis Center, and College of Chemistry, Zhengzhou University, Zhengzhou 450000, P. R. China.

[3]Department of Chemistry, The University of Sheffield, Sheffield S3 7HF

[4]School of Chemical Sciences, The University of Auckland, Auckland 1142, New Zealand.

[5]These authors contributed equally: Dafeng Zhang, Mengnan Li, Xue Yong.

*Corresponding authors.

E-mail: bzliu@hpu.edu.cn (Baozhong Liu), sylu2013@zzu.edu.cn (Siyu Lu)

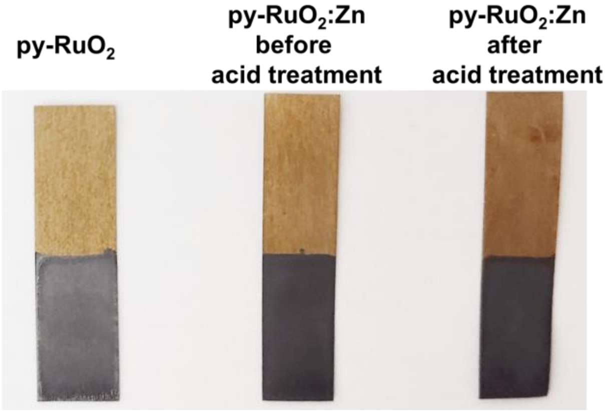

Supplementary Fig. 1 | Photographs of py-RuO_2, and py-RuO_2:Zn electrodes before and after the acid treatment.

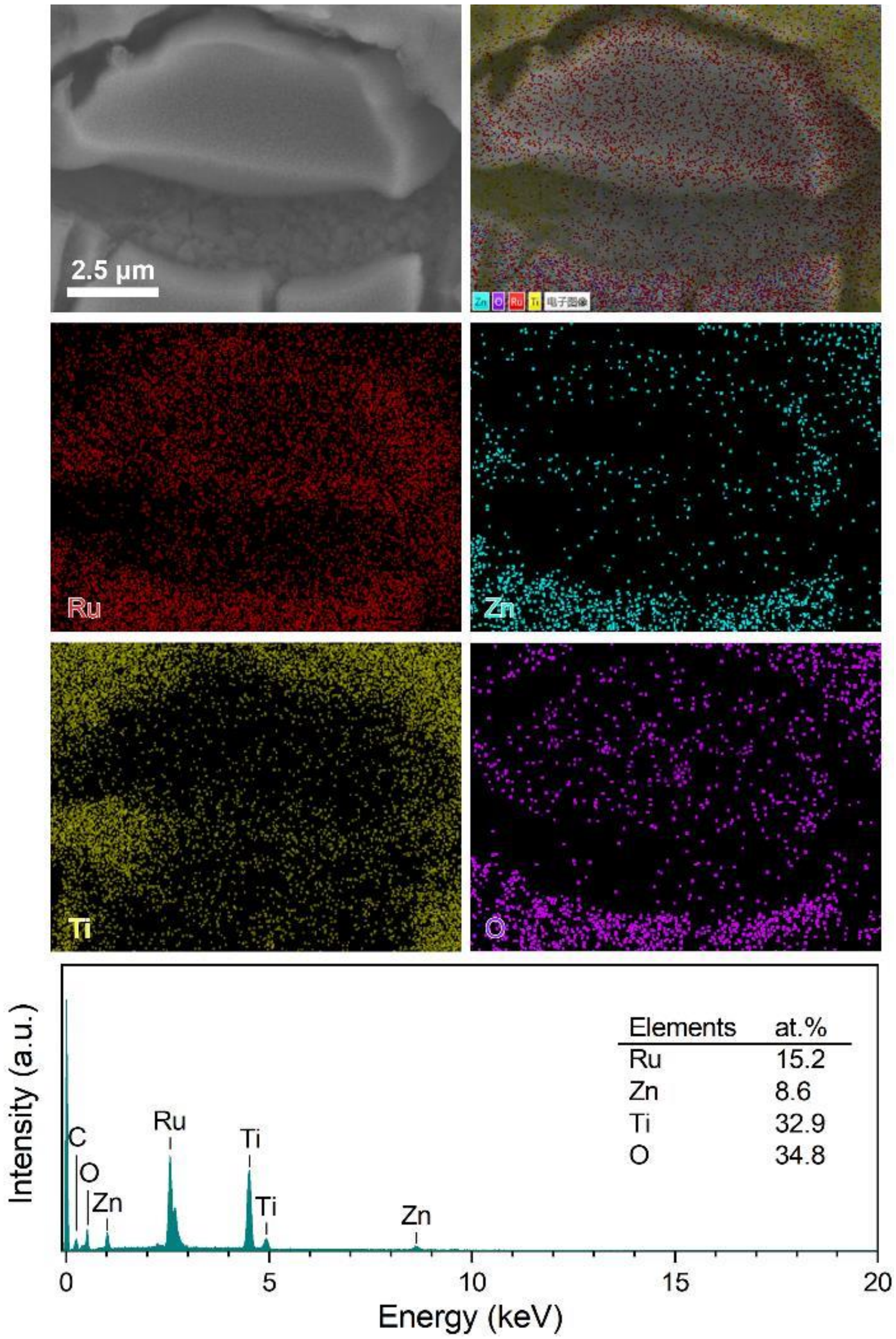

Supplementary Fig. 2 | SEM image (top left) and corresponding EDS maps for Ru, Zn, Ti, and O elements in py-RuO_2:Zn before the acid treatment. The atomic ratio of Zn:Ru is 0.57:1 determined from the EDS spectrum, close to the molar ratio in precursor (Zn:Ru = 0.5:1).

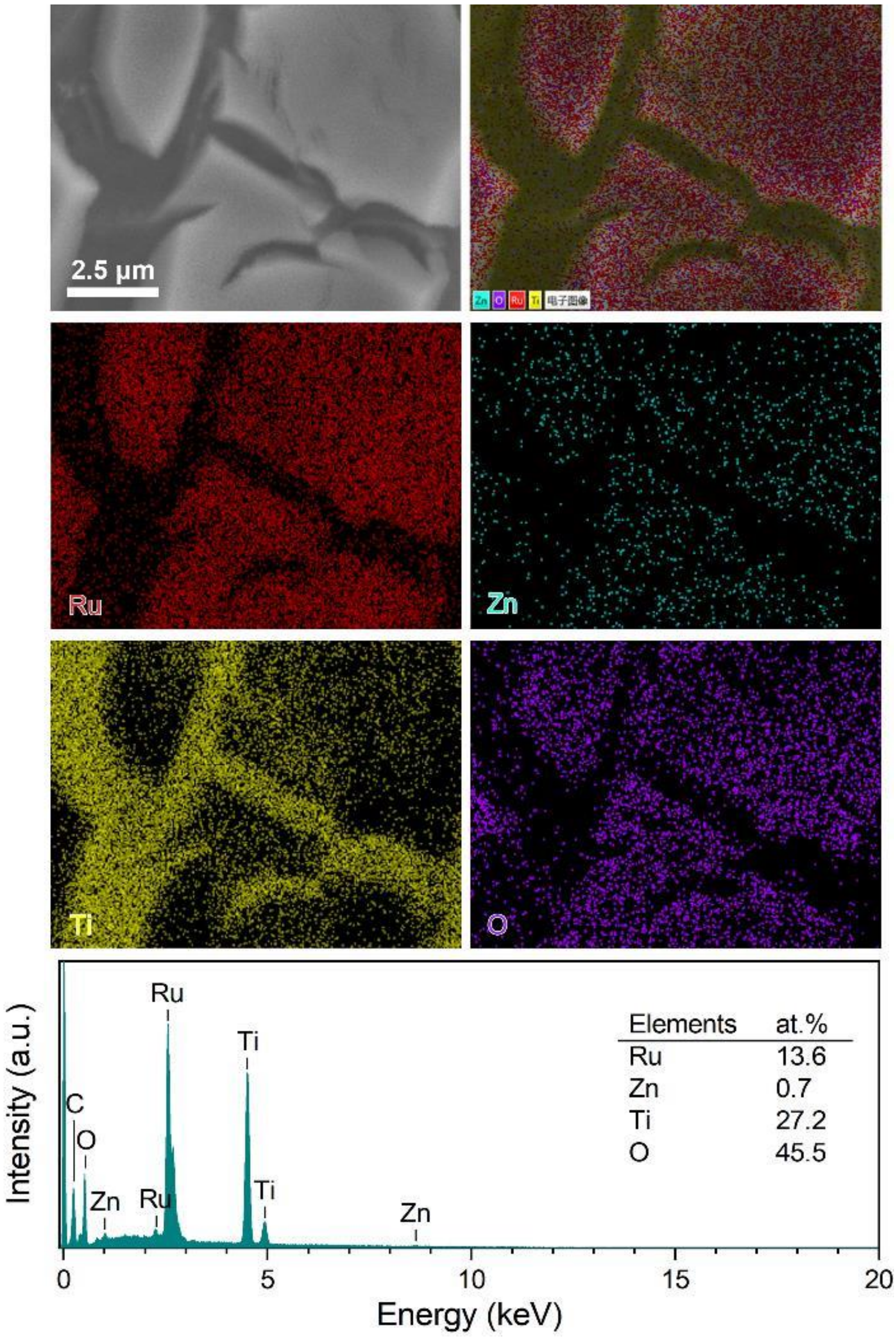

Supplementary Fig. 3 | SEM image (top left) and corresponding EDS maps for Ru, Zn, Ti, and O elements in py-RuO_2:Zn after the acid treatment. The atomic ratio of Zn:Ru is 0.0515:1 determined from EDS spectrum, close to the ICP-MS result (Zn:Ru = 0.0639:1).

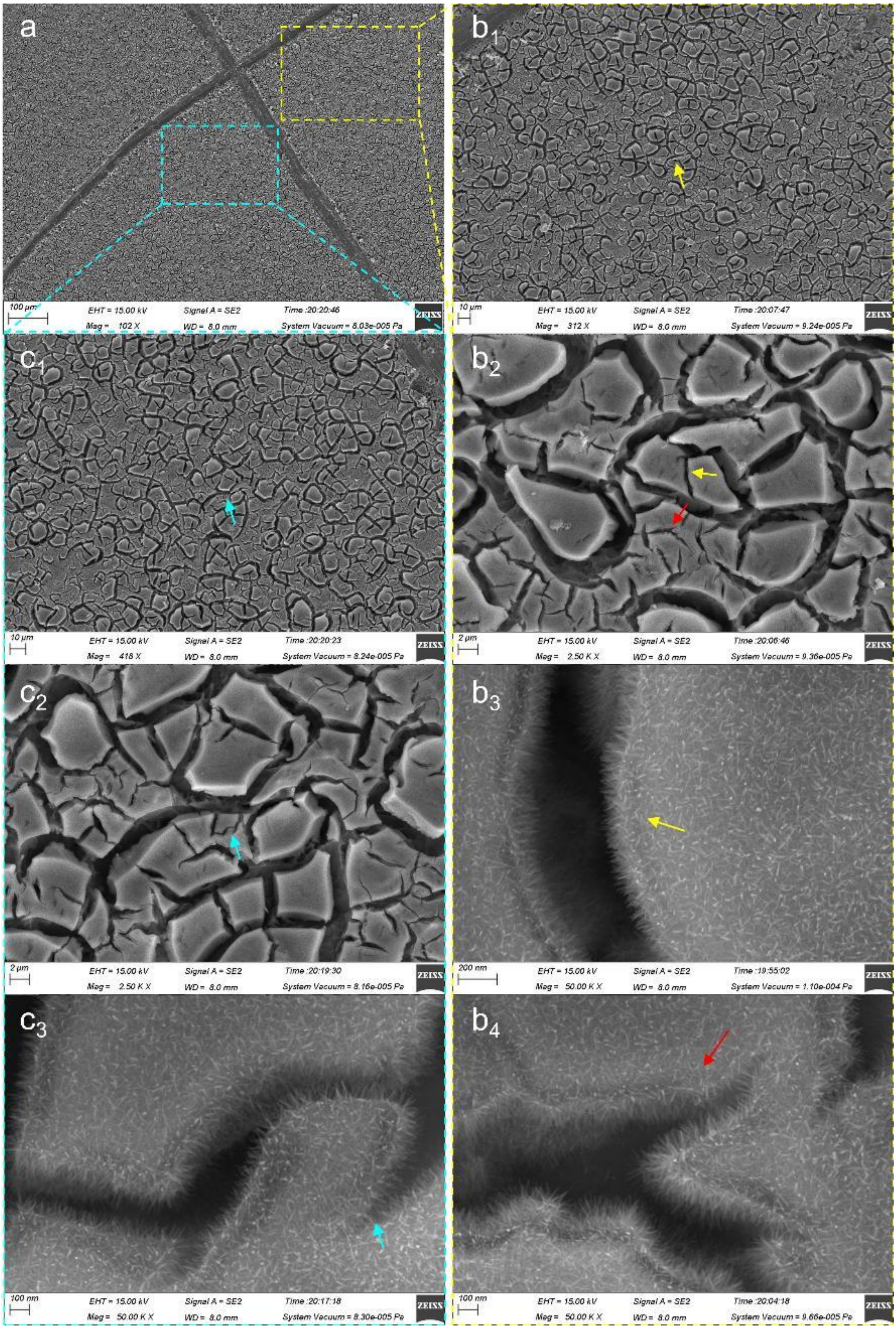

Supplementary Fig. 4 | SEM images of py-RuO_2:Zn.

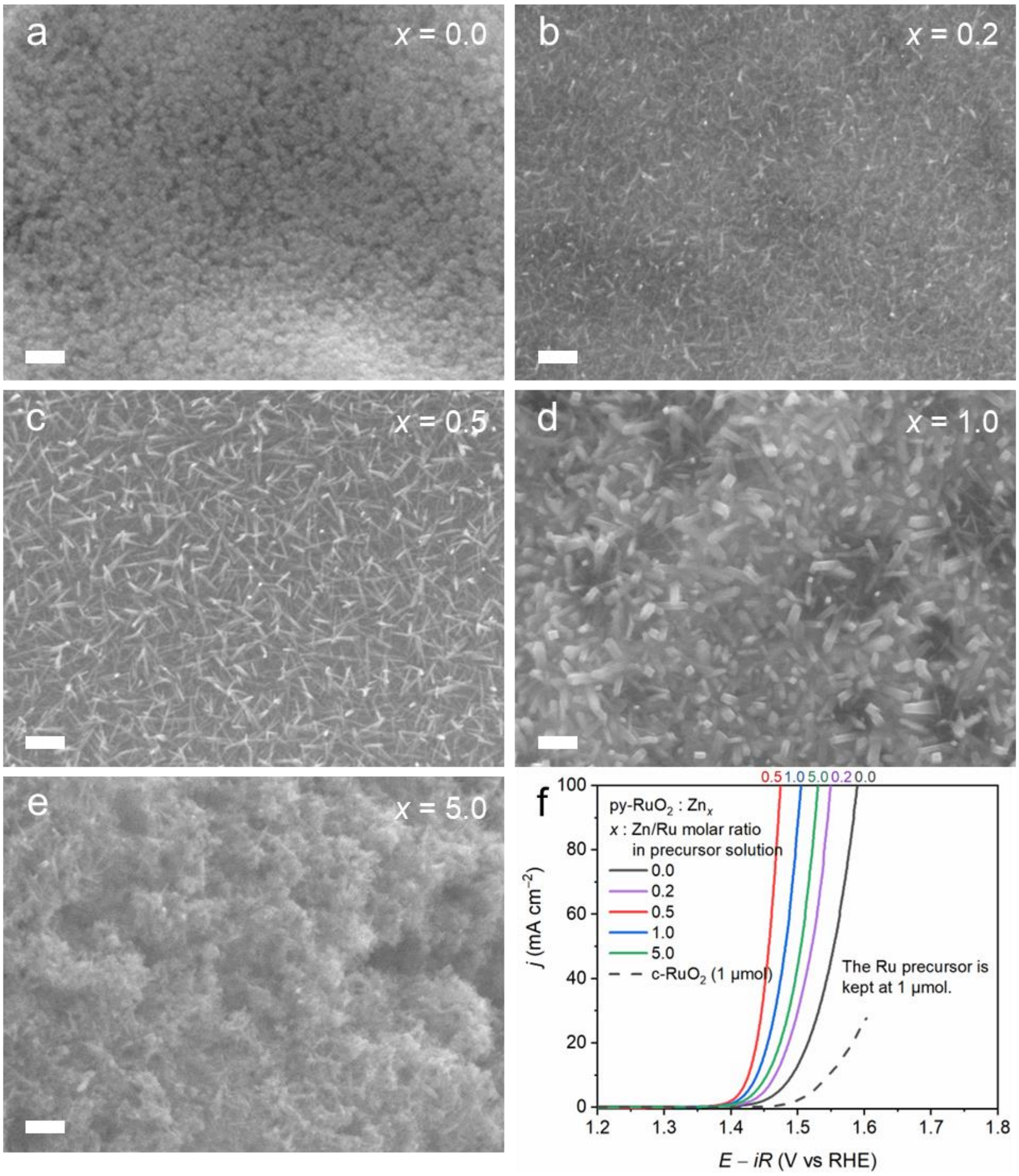

Supplementary Fig. 5 | SEM images and OER activity of py-RuO_2:Zn catalysts. (a–e) SEM images of py-RuO_2:Zn catalysts prepared using different amounts of Zn in the precursor solution. Scale bar: 100 nm. (f) LSV curves of different catalysts for OER in O_2-saturated H_2SO_4. The amount of Ru precursor was fixed at 1 μmol (electrode area: 0.5 cm^2).

The “x” in Supplementary Fig. 5f is referred to the Zn/Ru molar ratio in the precursor solution, equally to the dosage ratio of $Zn(NO_3)_2$ to $RuCl_3$ used in the catalyst preparation. The value is obviously higher than the final content of Zn dopants in py-RuO_2:Zn, due to the fact that most of the Zn elements from $Zn(NO_3)_2$ precursor were converted to the ZnO product under the pyrolytic treatment, rather than being doped into RuO_2 lattice. The unwanted ZnO composition was removed using an acid etching procedure before the subsequent experiments.

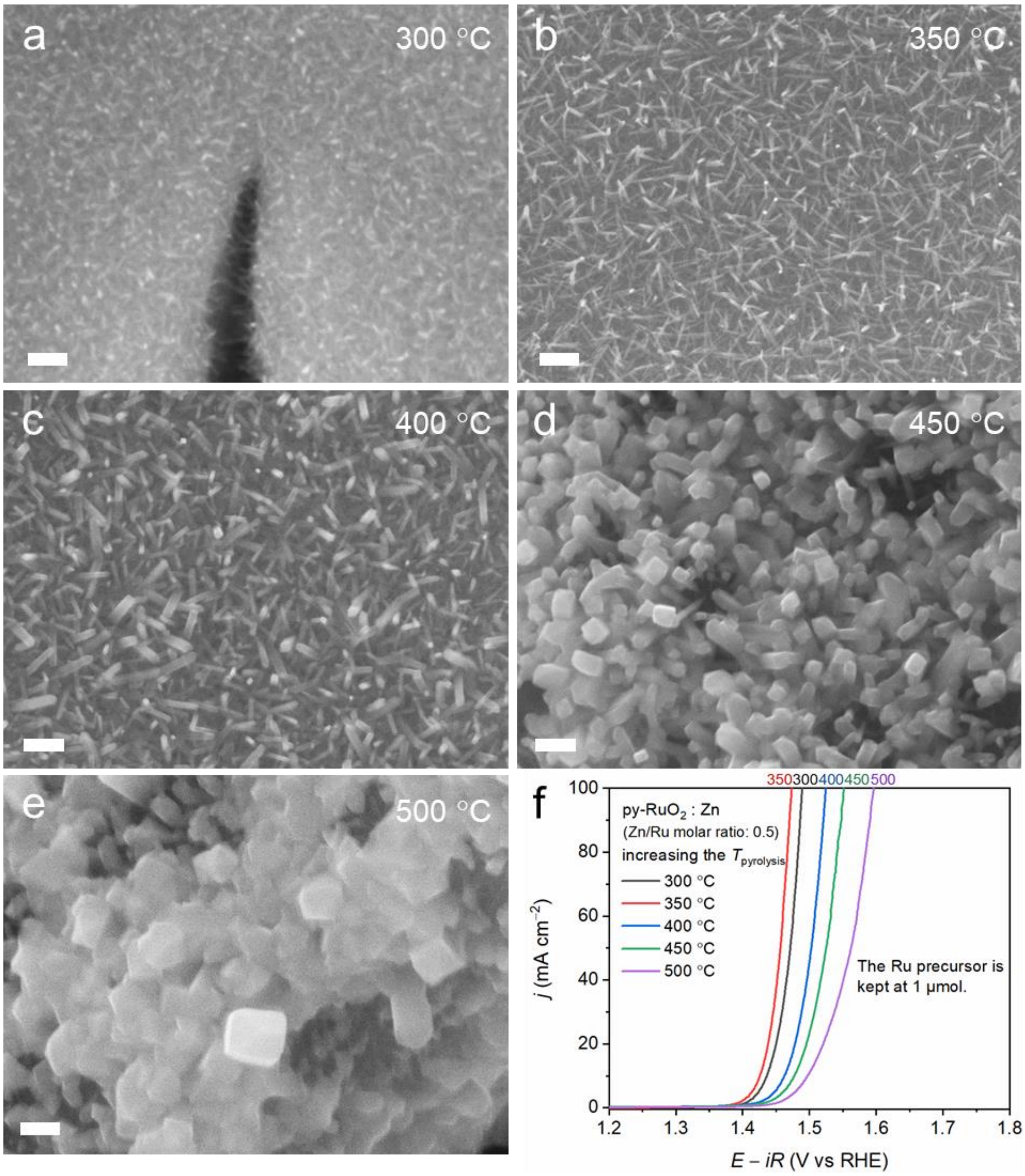

Supplementary Fig. 6 | SEM images and OER activity of py-RuO_2:Zn catalysts. (a–e) SEM images of py-RuO_2:Zn catalysts prepared at different pyrolysis temperatures. Scale bar: 100 nm. (f) LSV curves of different catalysts for OER in O_2-saturated 0.5 M H_2SO_4. The amount of Ru precursor was fixed at 1 μmol (electrode area: 0.5 cm^2) with a Zn/Ru molar ratio of 0.5.

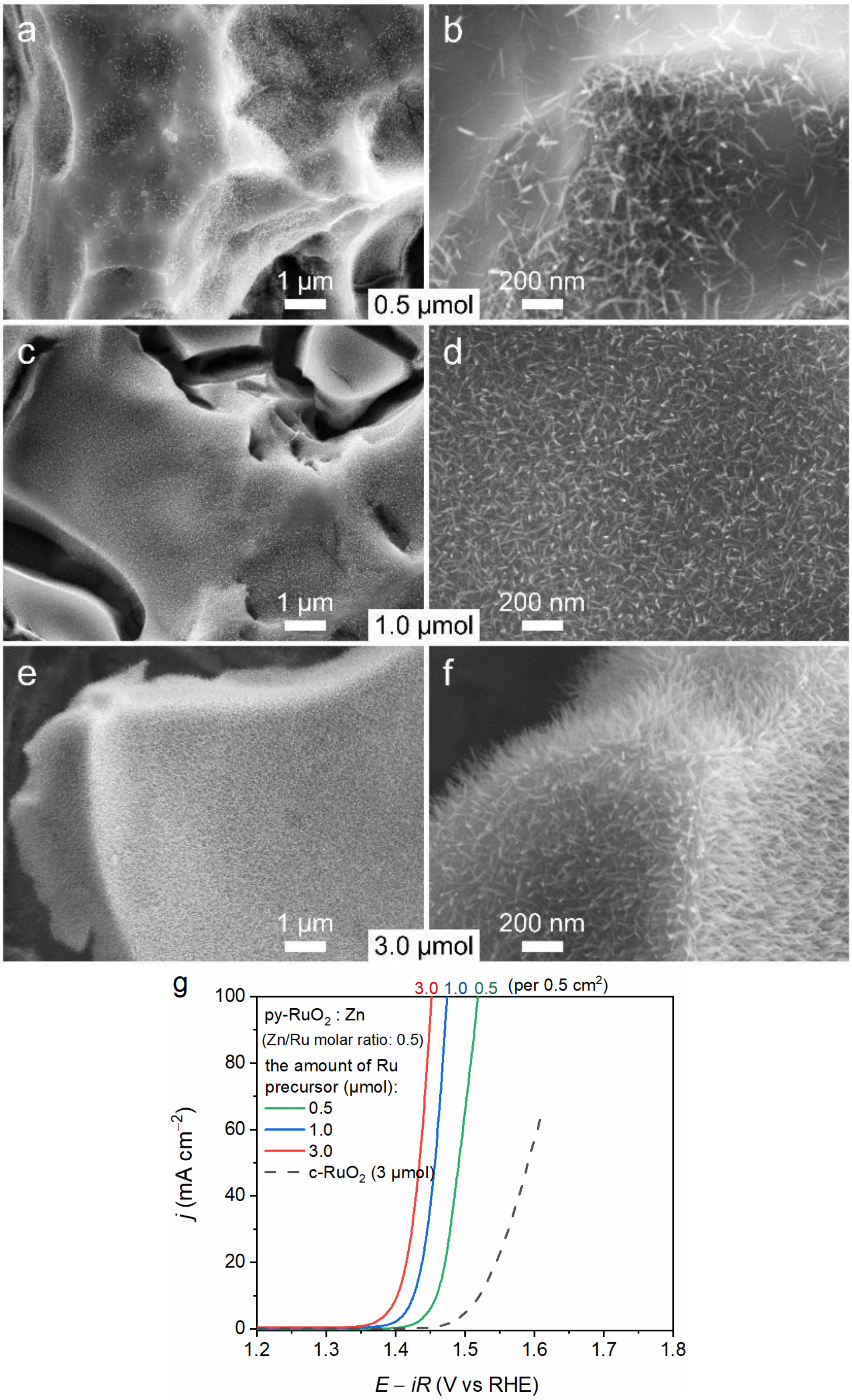

Supplementary Fig. 7 | SEM images and OER activity of py-RuO_2:Zn catalysts. (a–f) SEM images of py-RuO_2:Zn catalysts prepared using different amounts of Ru at a fixed Zn/Ru molar ratio of 0.5 in precursor solution (electrode area: 0.5 cm^2). (g) LSV curves of different catalysts for OER in O_2-saturated 0.5 M H_2SO_4.

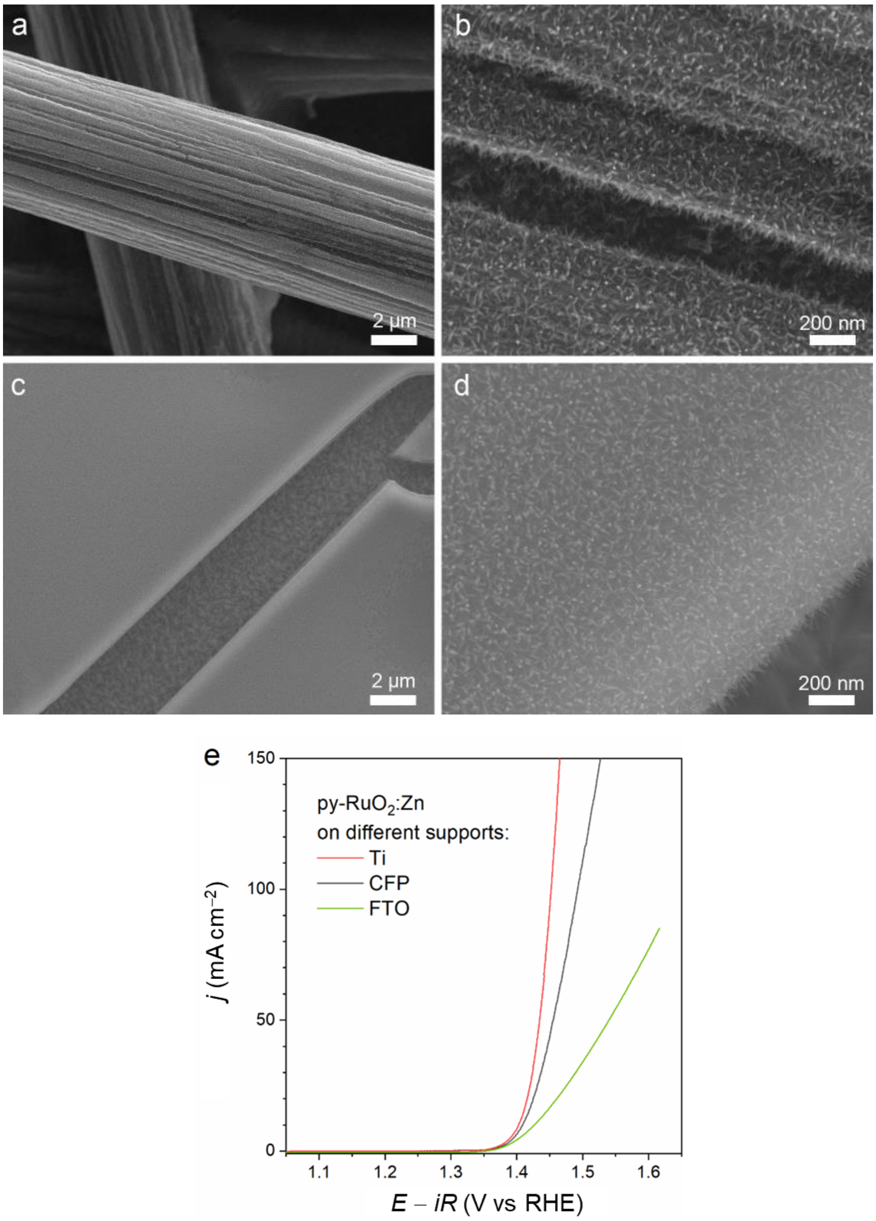

Supplementary Fig. 8 | SEM images and OER activity of py-RuO_2:Zn catalysts prepared on different substrates. SEM images of py-RuO_2:Zn nanowire arrays prepared on carbon fiber paper (CFP) (a, b) and fluorine-doped tin oxide (FTO) glass (c, d) under the same pyrolysis conditions. (e) LSV curves of py-RuO_2:Zn on Ti, CFP, and FTO glass supports for OER in 0.5 M H_2SO_4 solution with O_2 saturation.

Due to the harsh operation conditions of acidic OER, the CFP will undergo serious degradation. Although FTO glass is more stable under the corrosive reaction conditions, the relatively lower conductivity would hinder the electron transfer efficiency at the interface. Therefore, the Ti plate was selected as the support for py-RuO_2:Zn catalyst in this work, which is a widely used dimensionally stable anodes material in chlorine evolution process.

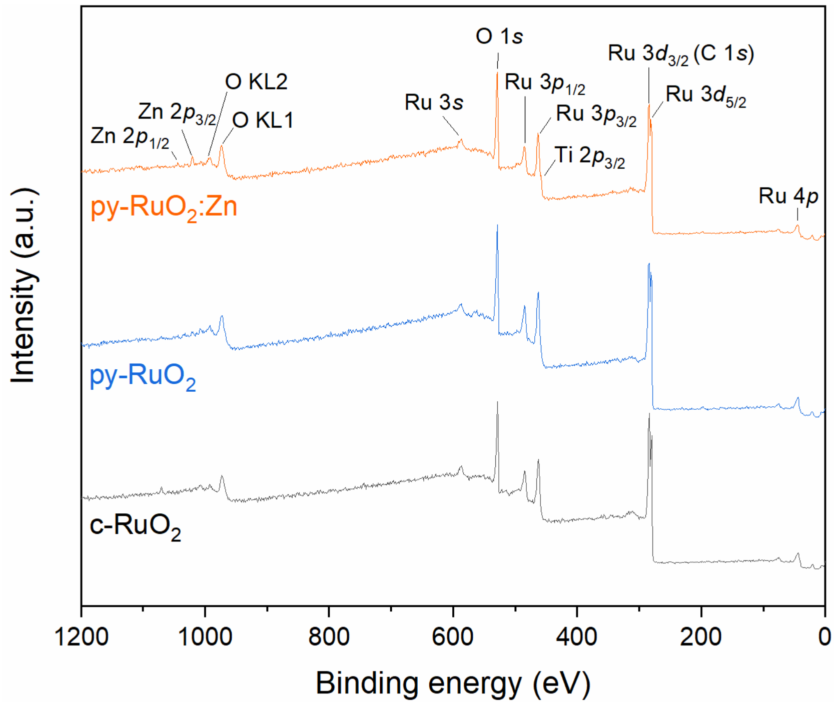

Supplementary Fig. 9 | XPS survey spectra for py-RuO$_2$:Zn, py-RuO$_2$ and c-RuO$_2$ catalysts.

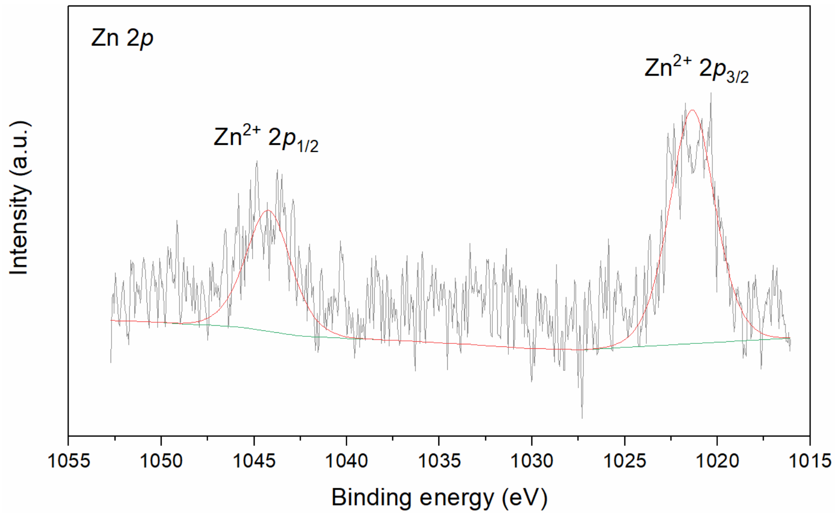

Supplementary Fig. 10 | Core-level Zn 2*p* XPS spectrum for the py-RuO$_2$:Zn catalyst.

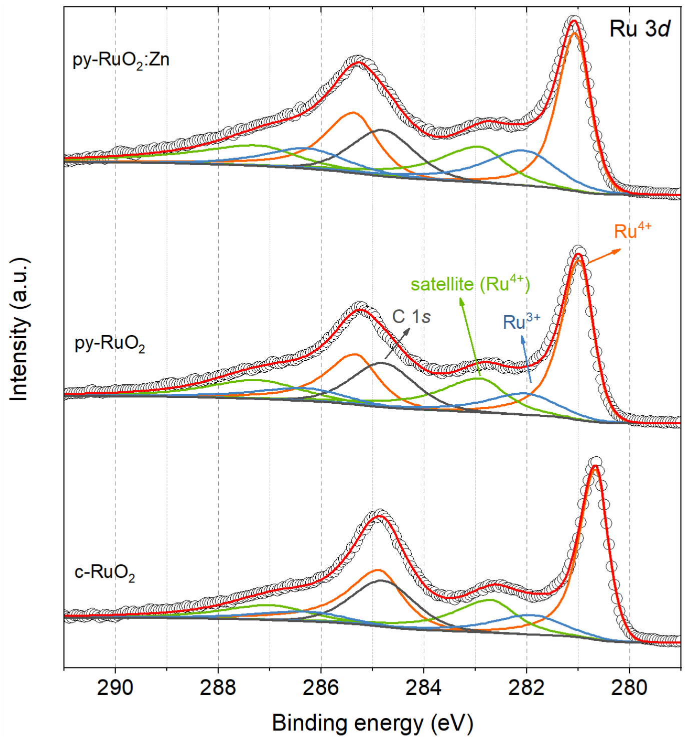

Supplementary Fig. 11 | Core-level Ru 3*d* XPS spectra for the py-RuO_2:Zn, py-RuO_2, and c-RuO_2 catalysts.

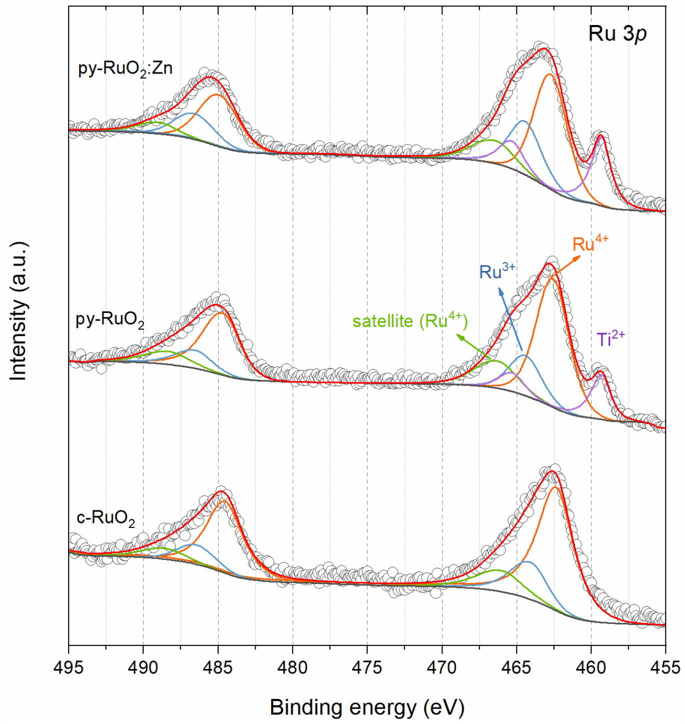

Supplementary Fig. 12 | Core-level Ru 3*p* XPS spectra for the py-RuO_2:Zn, py-RuO_2, and c-RuO_2 catalysts.

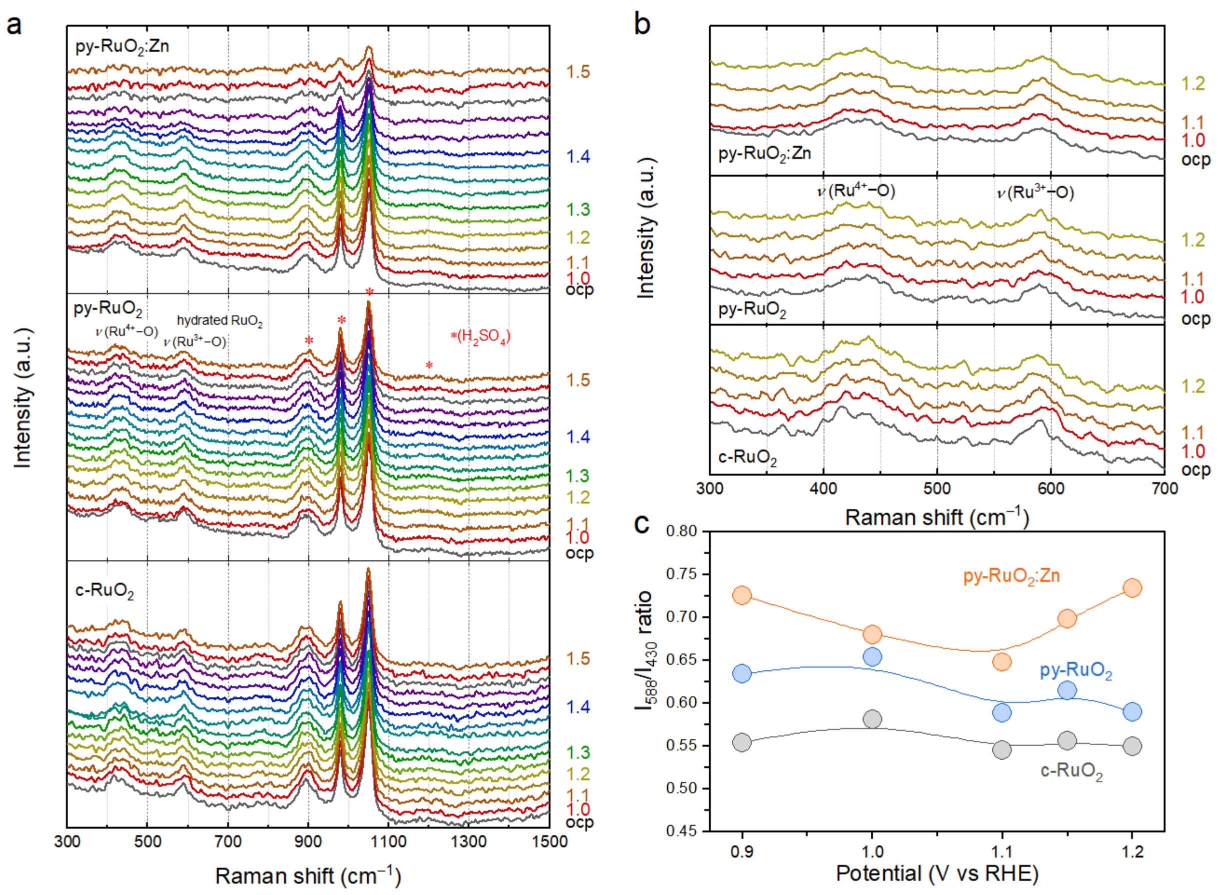

Supplementary Fig. 13 | In-situ Raman spectra of OER on the catalysts. (a, b) Raman spectra for py-RuO_2:Zn, py-RuO_2, and c-RuO_2 catalysts in O_2-saturated 0.5 M H_2SO_4 at given electrode potentials. (b) Normalized intensity of Raman band at 588 cm^{-1} to that at 430 cm^{-1} on the catalysts as a function of applied potential. The areas under the bands were used to calculate the I_{588}/I_{430} ratio.

Raman spectra were obtained at a Renishaw Raman Imaging Microscope System (inVia-Reflex) equipped with a CCD detector. Excitation radiation at 532 nm was used. The in-situ Raman spectroelectrochemical experiments were performed under constant cathodic potentials progressively stepping to positive limits from the open-circuit potential (OCP). Strong Raman bands at 430 and 588 cm^{-1} were observed on both py-RuO_2:Zn and c-RuO_2 catalysts, associated with the vibration of Ru^{4+}–O bonds and Ru^{3+}–O bonds of hydrated RuO_2 on the surface.[1] When further normalizing the intensity of the band at 588 cm^{-1} to that at 430 cm^{-1}, represented by the area ratio under the bands, we found that the py-RuO_2:Zn catalyst showed a higher intensity than two others, thereby possessing more Ru^{3+} species on the surface.

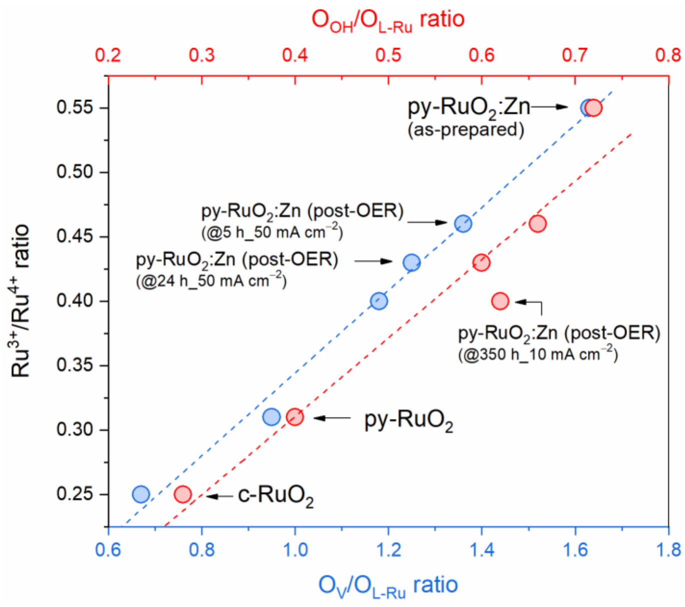

Supplementary Fig. 14 | Relationship analysis of Ru^{3+} abundance with the concentrations of V_O defects and OH adsorbates from the XPS results of Ru 3*p* and O 1*s*.

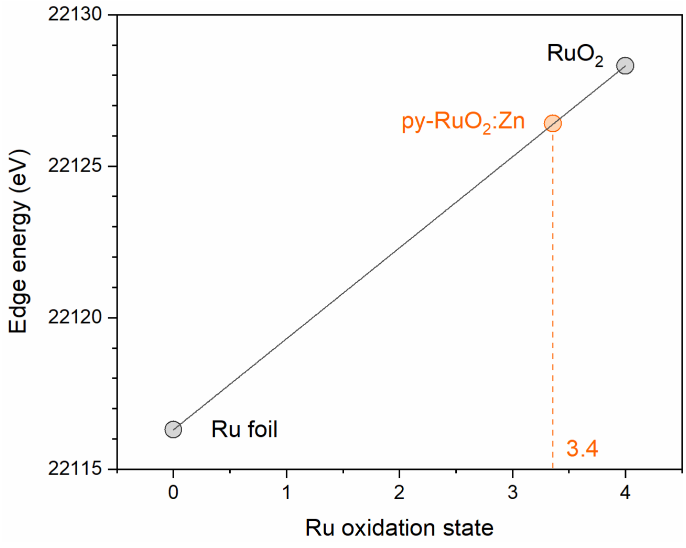

Supplementary Fig. 15 | The edge energies for Ru *K*-edge as a function of the oxidation state of the Ru.

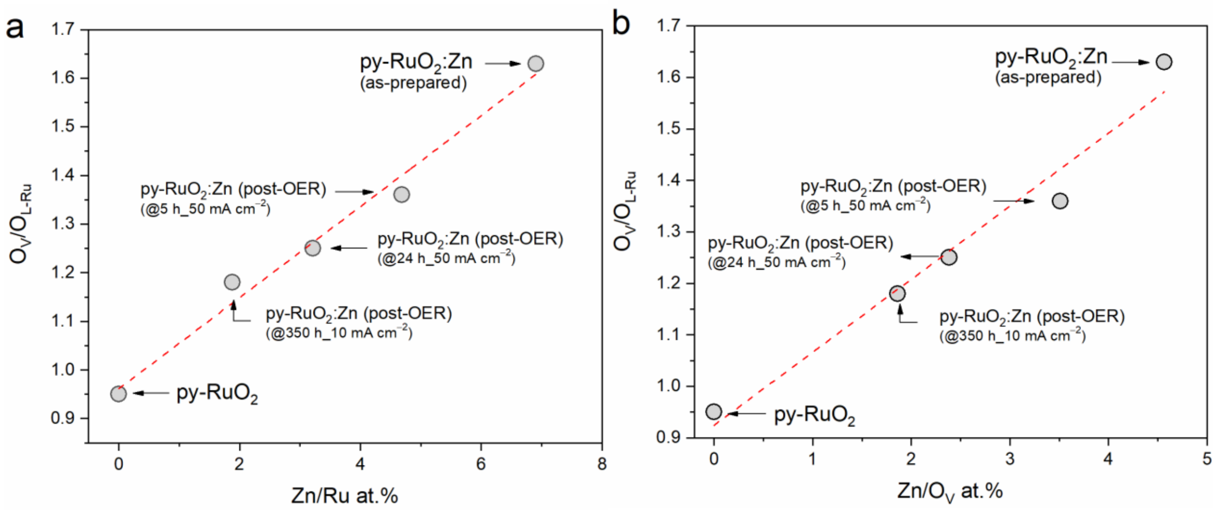

Supplementary Fig. 16 | The dependence of V_O defect ($O_V/O_{L\text{-}Ru}$ ratio) on the content of Zn dopants on the basis of XPS results. The content of Zn dopants was respectively represented by (a) Zn/Ru at.% and (b) Zn/O_V at.% values.

There was a linear dependance of $O_V/O_{L\text{-}Ru}$ on the Zn content, indicating that the doping of Zn element can induce the generation of V_O defects. However, a longitudinal interception of the fitted curve in Supplementary Fig. 16a was found to be 0.96. This means that the undoped RuO_2 would also contain high concentration of V_O defects. The value, interestingly, well matched the measured 0.97 for $O_V/O_{L\text{-}Ru}$ on the undoped py-RuO_2 catalyst prepared here. The presence of V_O defect in pure RuO_2 was caused by the relatively lower calcination temperature of catalyst synthesis procedure.[2] In the meantime, the doping of Zn induced a further linear increase of V_O defect, from 0.97 to 1.63 of $O_V/O_{L\text{-}Ru}$ as the Zn/Ru at.% increased from 0 to 10.4% on the surface of the py-RuO_2:Zn catalysts. Accordingly, two factors, Zn doping and catalyst synthesis method, have resulted in the generation of V_O defect.

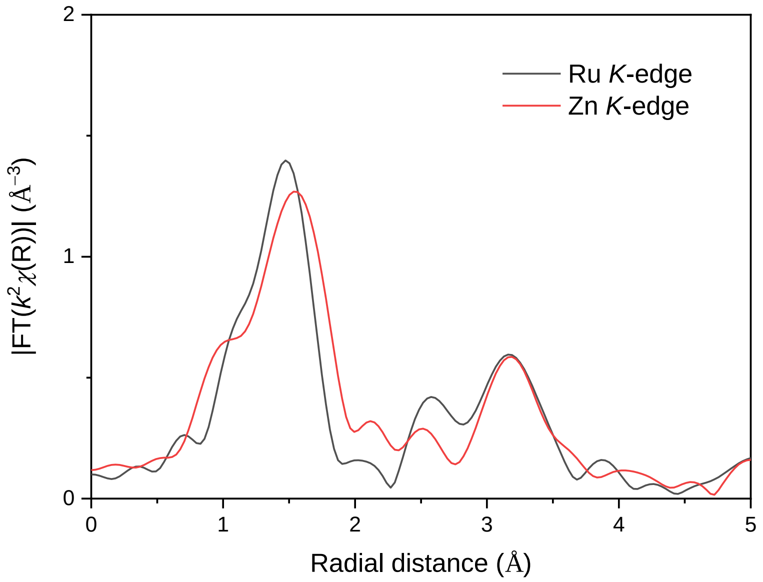

Supplementary Fig. 17 | Ru *K*-edge and Zn *K*-edge FT-EXAFS R-space spectra for py-RuO_2:Zn.

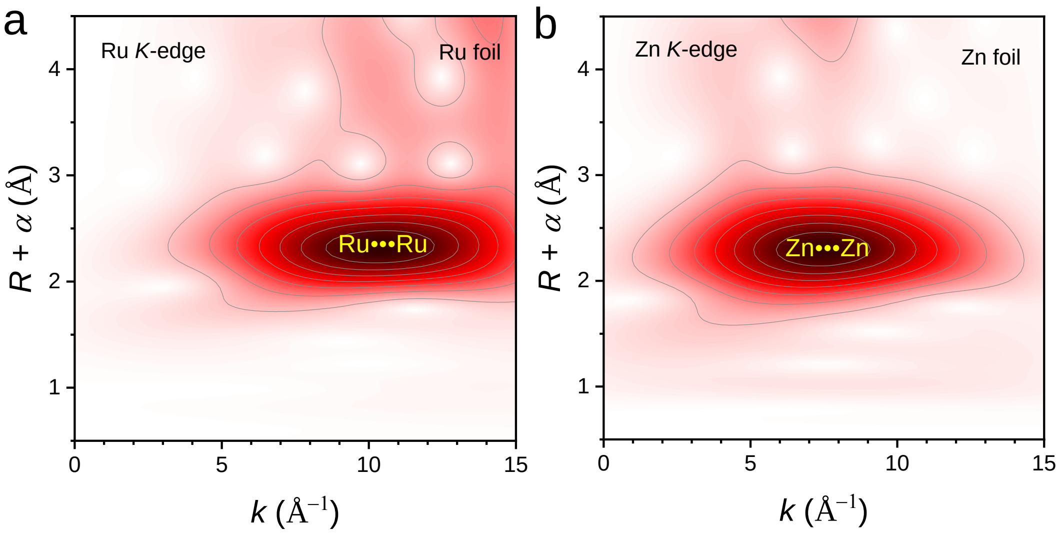

Supplementary Fig. 18 | WT-EXAFS spectra for metallic Ru and Zn. (a) Ru *K*-edge and (b) Zn *K*-edge WT-EXAFS spectra for Ru foil and Zn foil, respectively.

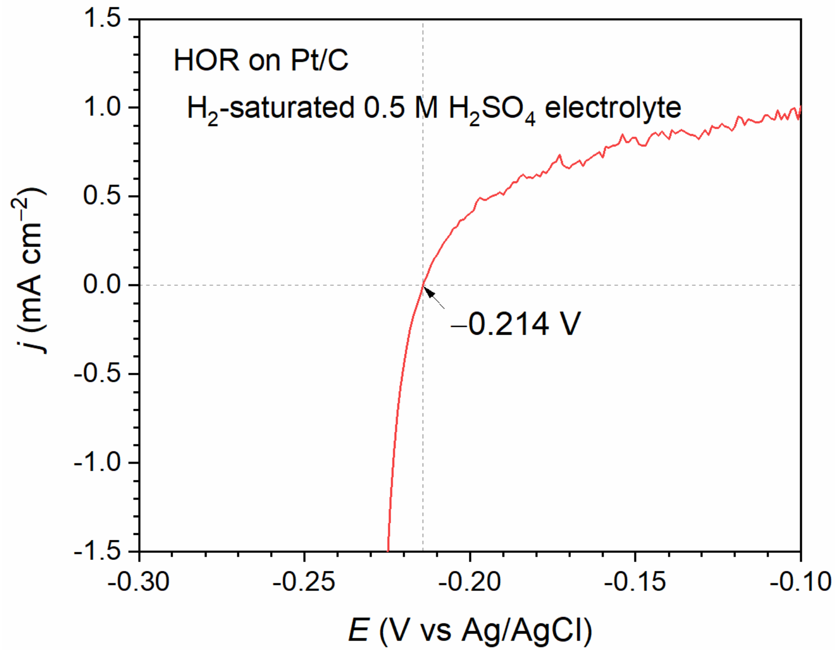

Supplementary Fig. 19 | LSV curve for the calibration of Ag/AgCl electrode in 0.5 M H_2SO_4 electrolyte with H_2 saturation. Commercial Pt/C, Ag/AgCl, and Pt plate were used as working, reference, and counter electrodes, respectively. scan rate: 1 mV s^{-1}.

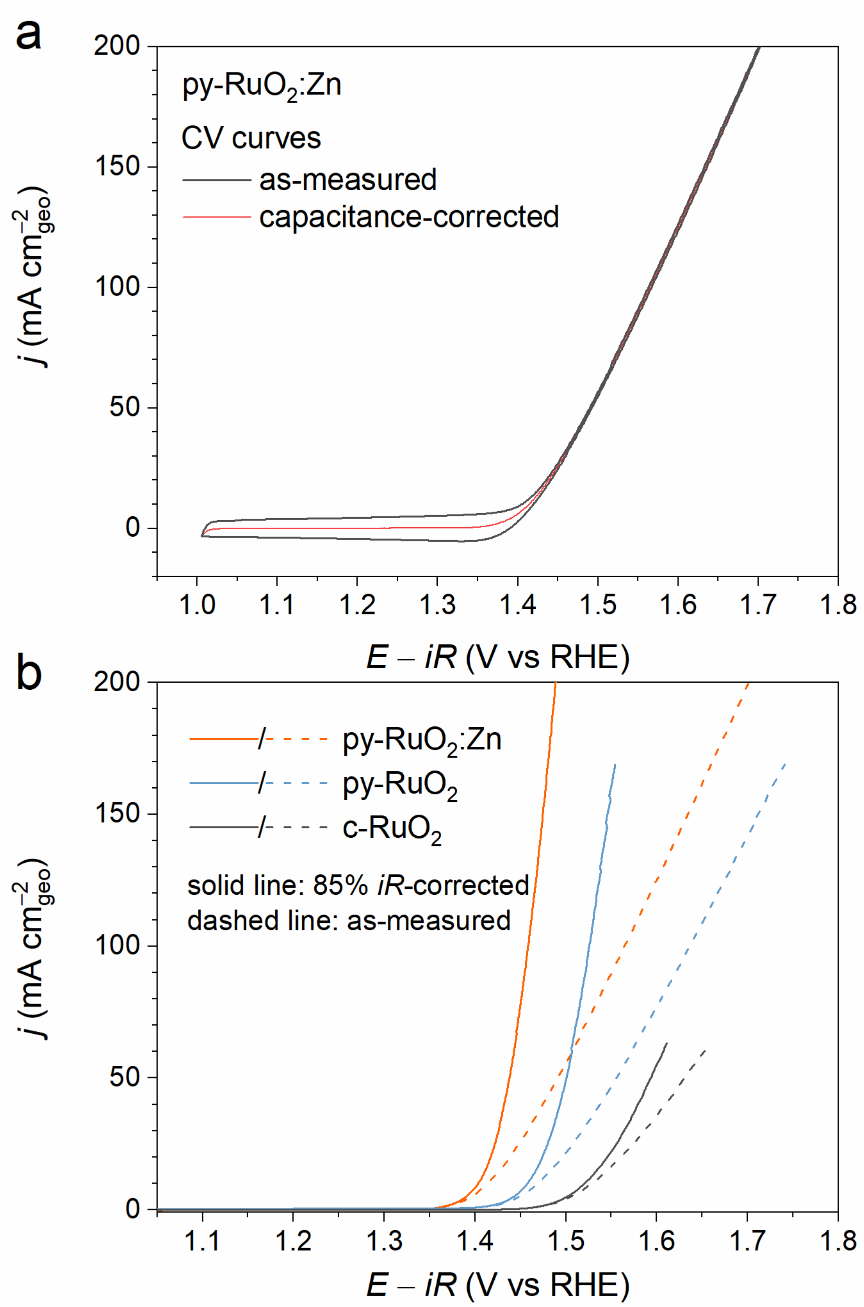

Supplementary Fig. 20 | Capacitance-correction and *iR*-correction of CV curve for OER. (a) Capacitance-correction of CV curve for OER on the py-RuO_2:Zn catalyst in O_2-saturated 0.5 M H_2SO_4 solution. (b) As-measured and 85% *iR*-corrected LSV curves for OER on the py-RuO_2:Zn, py-RuO_2, and c-RuO_2 catalysts in O_2-saturated 0.5 M H_2SO_4 solution. Solution resistances for *iR*-correction are 2.8, 2.6, and 4.5 Ω for py-RuO_2:Zn, py-RuO_2, and c-RuO_2, respectively.

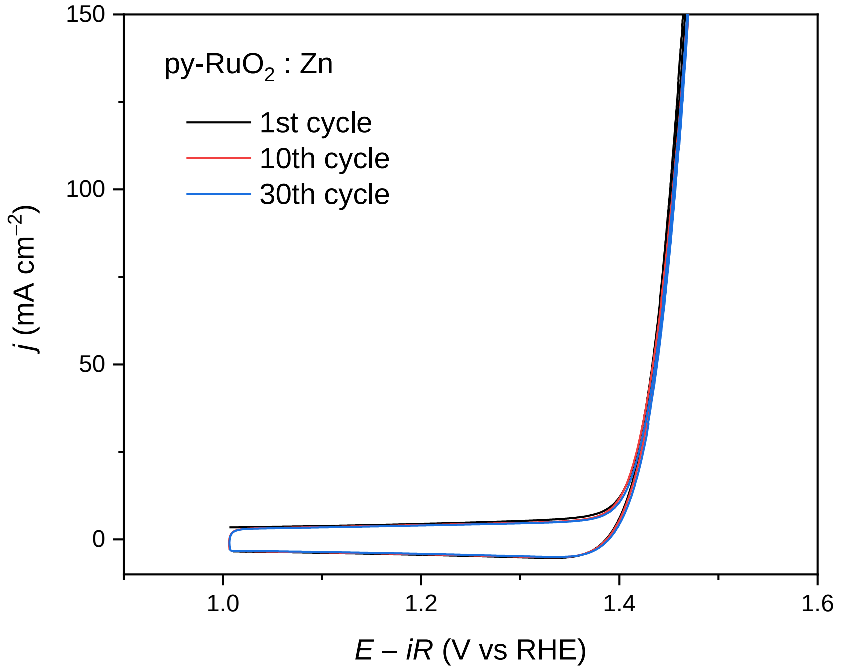

Supplementary Fig. 21 | A comparison of CV curves for OER during initial 30 cycles on the py-RuO_2:Zn catalyst in O_2-saturated 0.5 M H_2SO_4 solution.

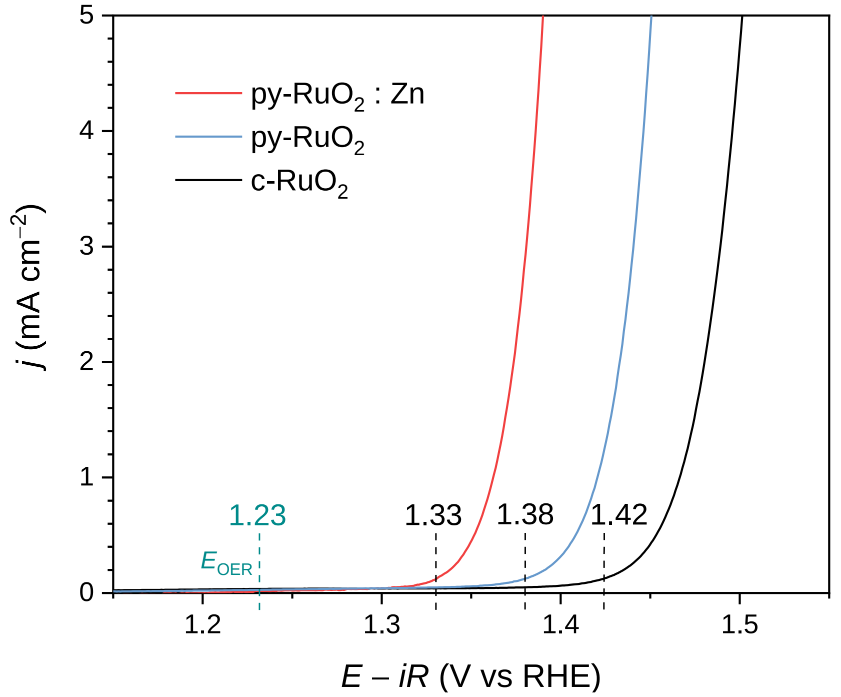

Supplementary Fig. 22 | Onset potential analysis for OER. LSV curves for OER on the py-RuO_2:Zn, py-RuO_2, and c-RuO_2 catalysts in O_2-saturated 0.5 M H_2SO_4 solution. The onset potentials for OER on different catalysts are indicated.

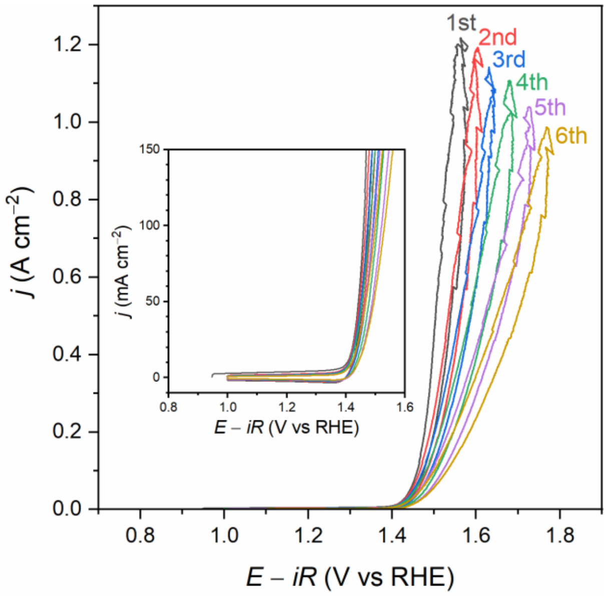

Supplementary Fig. 23 | Continous CV cycling curves of OER on py-RuO2:Zn. Six continous CV cycling curves of OER on the py-RuO_2:Zn catalyst under the condition when the current density reached higher than 1 A cm^{-2}. No capacitance correction was performed on the CV curves.

Clearly, the current density decayed significantly as the test progressed, most probabaly due to the degradation of the catalyst under the high current density. An obvious hysteresis between the anodic and cathodic curves was further observed. also probably caused by the degradation of the catalyst under the high current density.

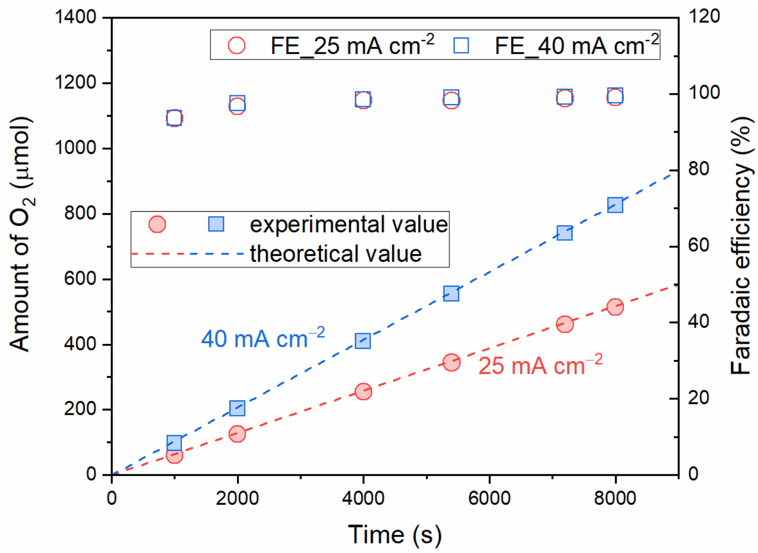

Supplementary Fig. 24 | Faradaic efficiency analysis for OER on py-RuO2:Zn. The FE of OER on py-RuO2:Zn catalyst as a function of reaction time determined by the water displacement method at current densities of 25 and 40 mA cm^{-2}. The geometric area of electrode was 1.0 cm^2.

Faradaic efficiency (FE) of OER was determined by water displacement method. The FE was calculated by the equation (1):

$$FE(\%) = \frac{n_{exp}(O_2)}{n_{theor}(O_2)} \times 100 \quad (1)$$

where $n_{exp}(O_2)$ and $n_{theor}(O_2)$ are experimental and theoretical amount of O_2 produced during the OER process.

According to the Faraday's law, the $n_{theor}(O_2)$ was calculated by the equation (2):

$$n_{thero}(O_2) = \frac{I \cdot t}{z \cdot F} \text{ (mol)} \quad (2)$$

where I (A) is the current, t (s) is the OER reaction time, z = 4 is the electron transfer number of OER, F = 96485 C mol^{-1} is the Faraday constant.

The was determined by a water displacement method and calculated by the ideal gas law:

$$n_{exp}(O_2) = \frac{p \cdot V}{R \cdot T} \text{ (mol)} \quad (3)$$

where p = (101325 – 2813) Pa is the partial pressure of O_2 produced, V (m^3) is the volume of O_2 produced, R = 8.314 J mol^{-1} K^{-1} is the ideal gas constant, T = 293.15 K is the reaction temperature.

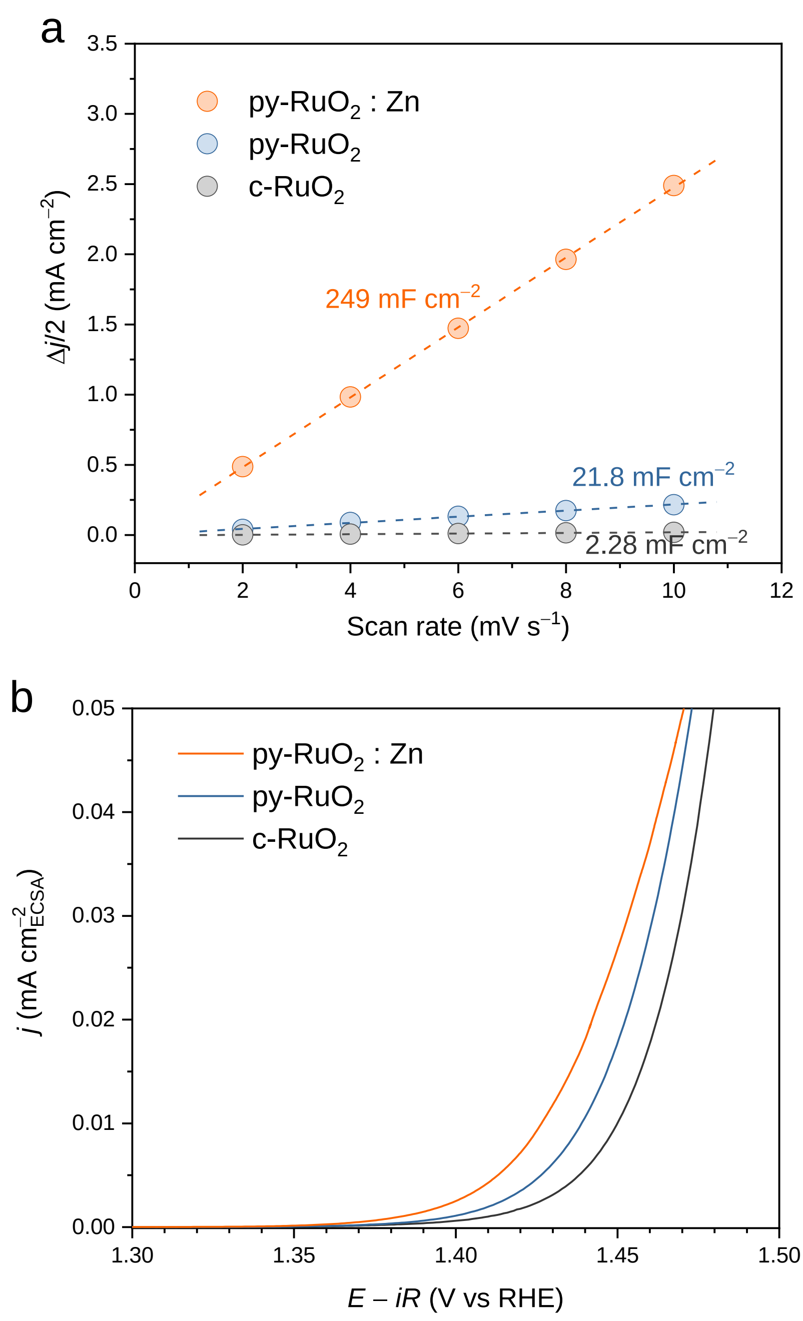

Supplementary Fig. 25 | C_{dl} values and ECSA normalized LSV curves for OER on different catalysts. (a) C_{dl} values for OER on the py-RuO_2:Zn, py-RuO_2, and c-RuO_2 catalysts in O_2-saturated 0.5 M H_2SO_4 solution. (b) ECSA normalized LSV curves for acidic OER on different catalysts. The ECSA values were calculated by dividing the C_{dl} by the capacitance of an ideal planar metal oxides with smooth surface (60 µF cm^{-2}).

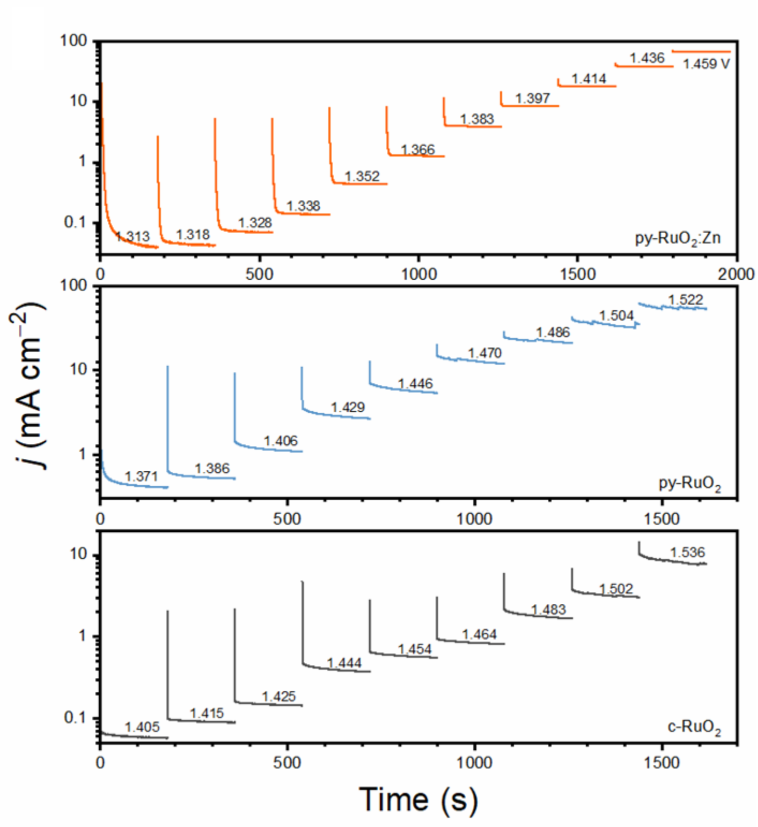

Supplementary Fig. 26 | Chronoamperometric responses of the py-RuO$_2$:Zn, py-RuO$_2$, and c-RuO$_2$ catalysts for OER at different potentials.

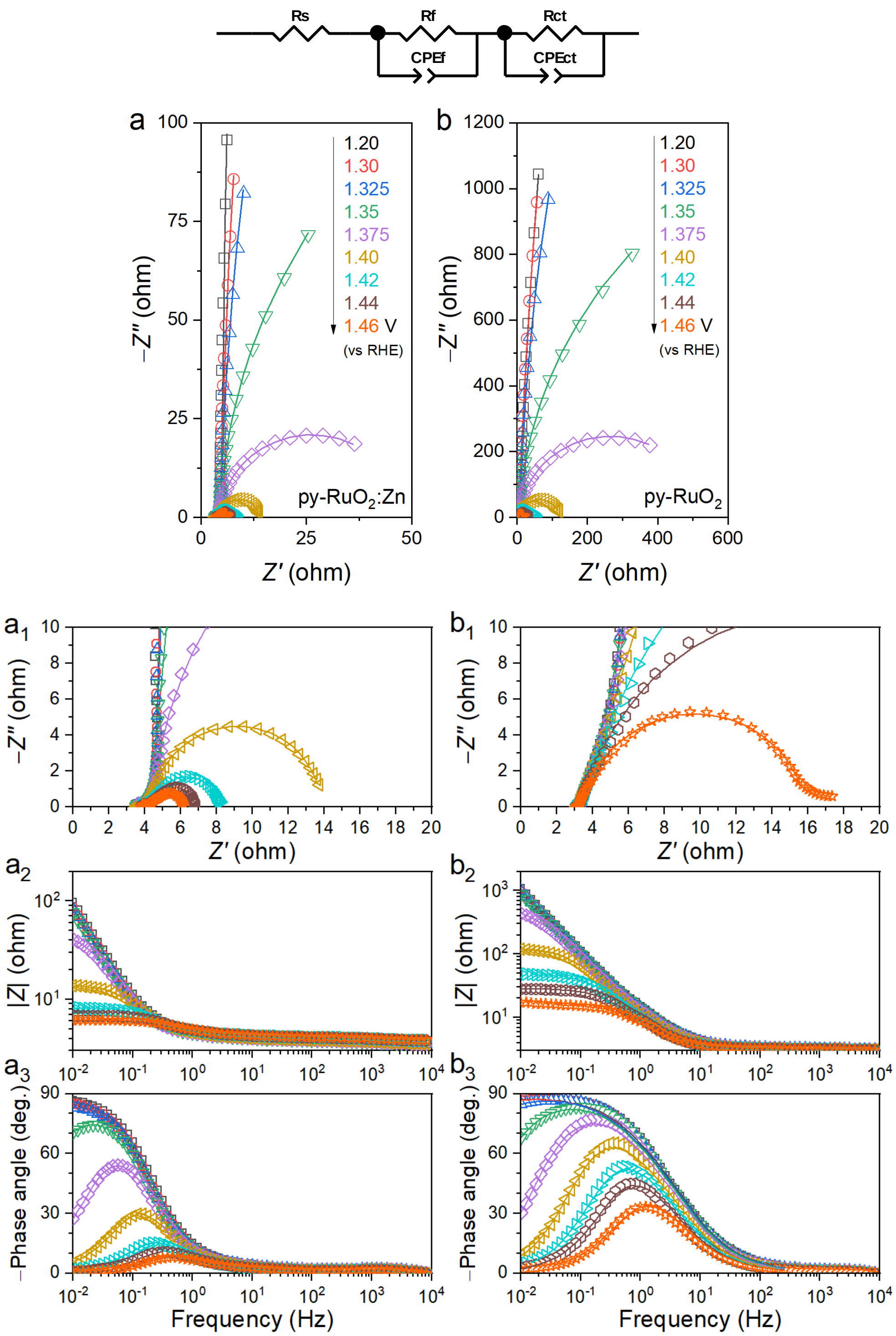

Supplementary Fig. 27 | EIS analysis for OER on different catalysts. EIS results for OER on the (a, a_1–a_3) py-RuO_2:Zn and (a, a_1–a_3) py-RuO_2 catalysts in O_2-saturated 0.5 M H_2SO_4. (a–a_1, b–b_1) Nyquist and (a_2–a_3, b_2–b_3) Bode plots at different potentials. EIS tests were performed at different applied potentials versus RHE (without *iR*-compensation) in the frequency range of 0.01 Hz to100 KHz.

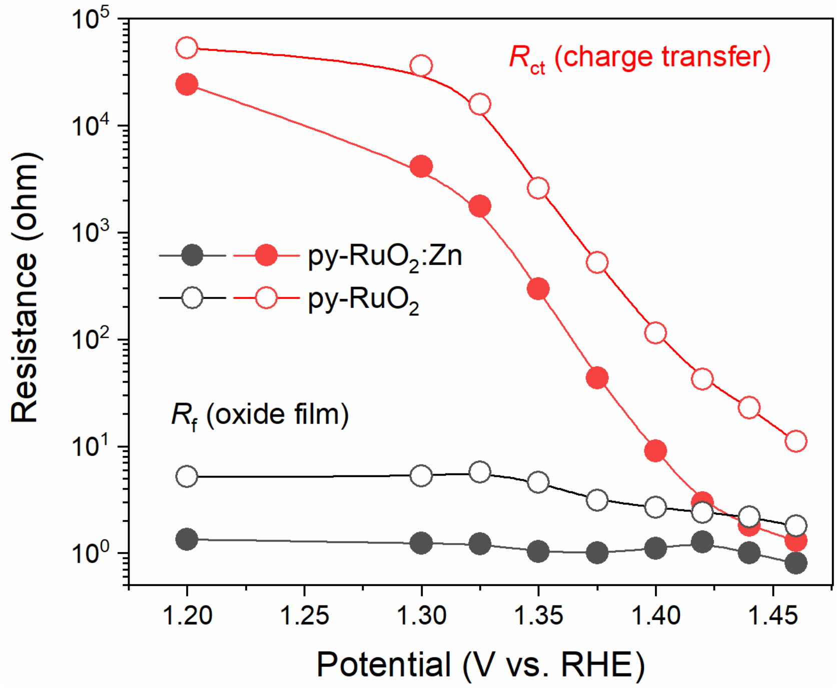

Supplementary Fig. 28 | Resistance analysis for OER on different catalysts. Charge transfer resistances (R_{ct}) and oxide film resistances (R_f) at different potentials for OER on the py-RuO_2:Zn and py-RuO_2 catalysts were measured in O_2-saturated 0.5 M H_2SO_4 solution. The resistance values were calculated by fitting the EIS spectra shown in Supplementary Fig. 27.

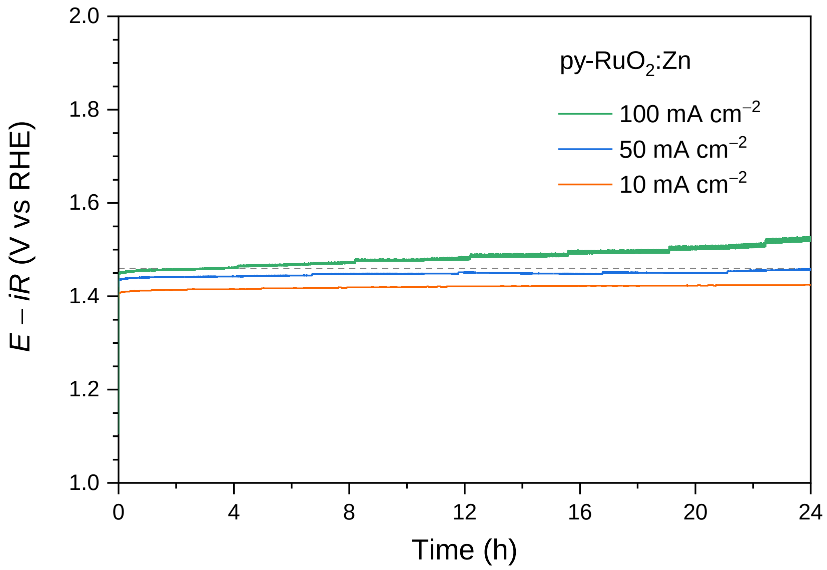

Supplementary Fig. 29 | Chronopotentiometric stability tests of py-RuO$_2$:Zn for OER at 10, 50, and 100 mA cm^{-2} for 24 h in 0.5 M H_2SO_4. The dashed line was assigned to a potential of 1.46 V.

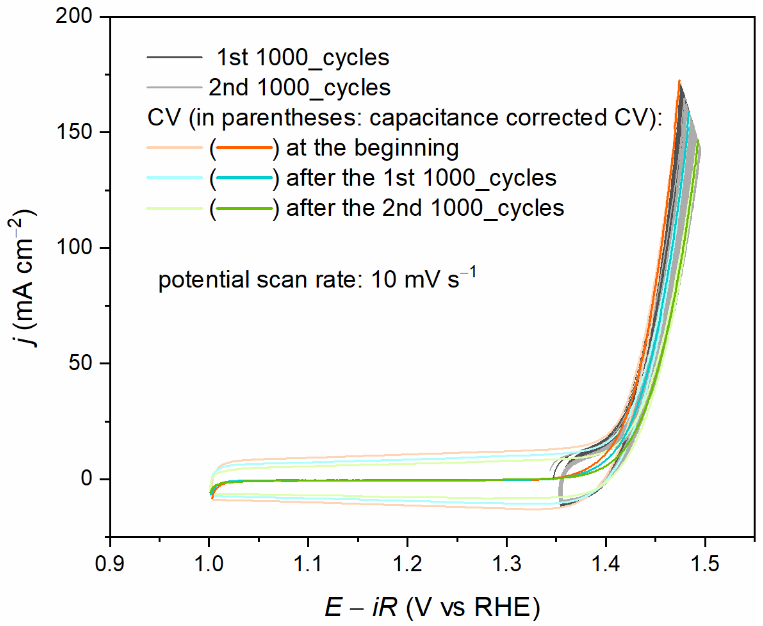

Suppementary Fig. 30 | Durability test of the py-RuO$_2$:Zn catalyst for acidic OER under the CV condition up to 2000 cycles. A doubled catalyst loading, about 520 mg per 0.5 cm^2, was adopted for this measurement. Potential scan rate was 10 mV s^{-1}.

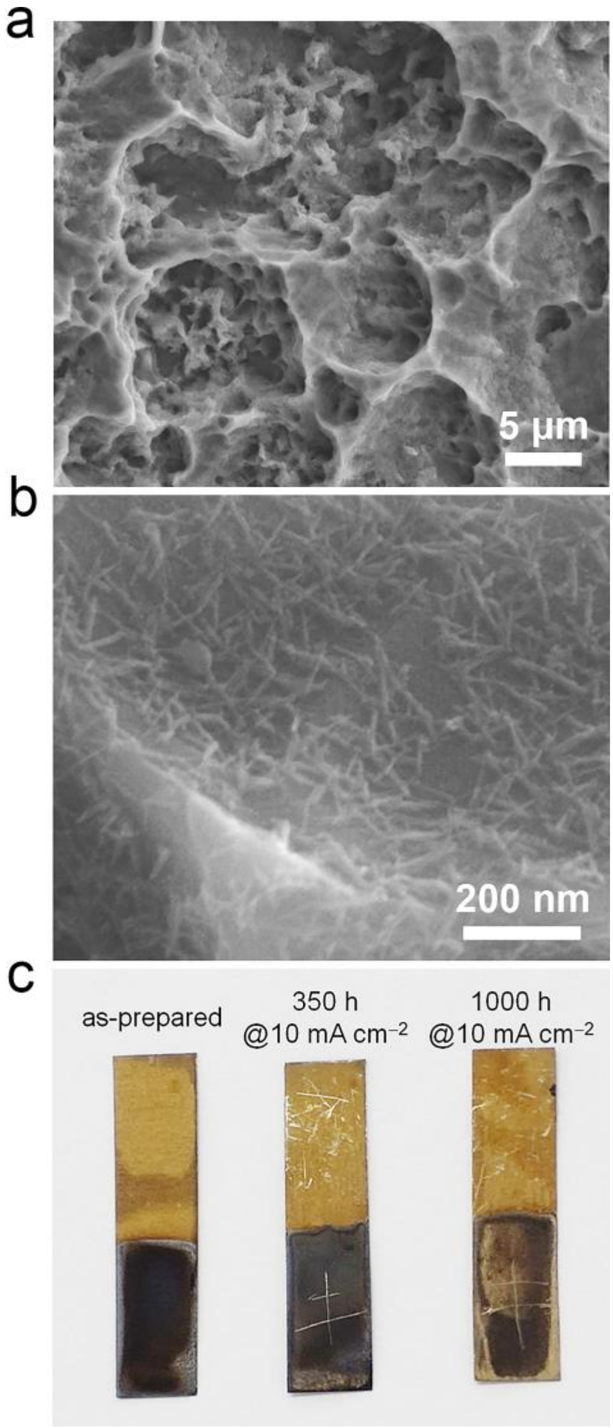

Supplementary Fig. 31 | SEM images and optical photograph of catalysts. (a, b) SEM images of the py-RuO_2:Zn catalyst after an OER stability test at 10 mA cm^{-2} for 1000 h. (c) Optical photograph of py-RuO_2:Zn electrode before and after the stability tests.

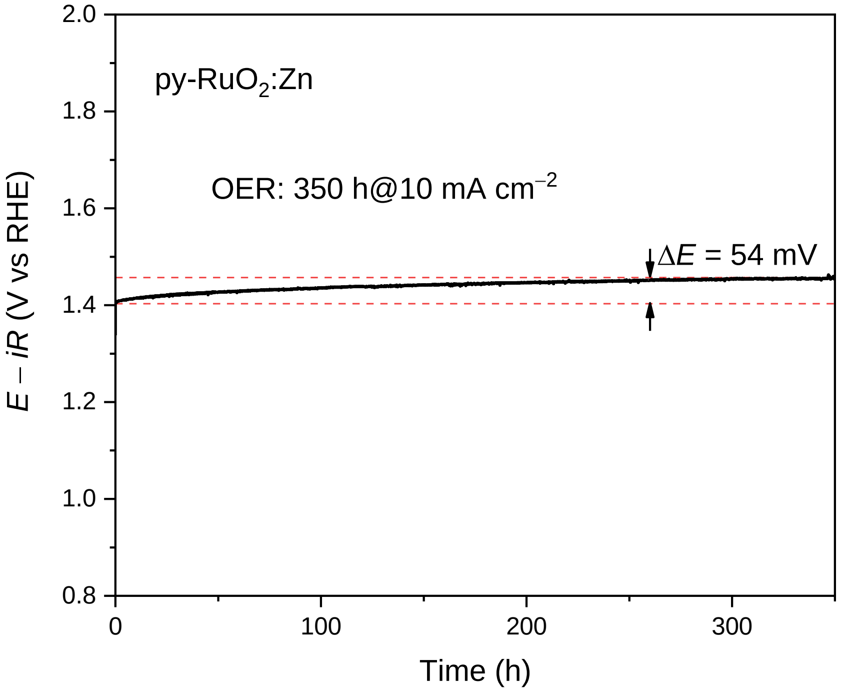

Supplementary Fig. 32 | CP curve for OER stability test on the py-RuO_2:Zn catalyst at 10 mA cm^{-2} for 350 h in 0.5 M H_2SO_4 solution.

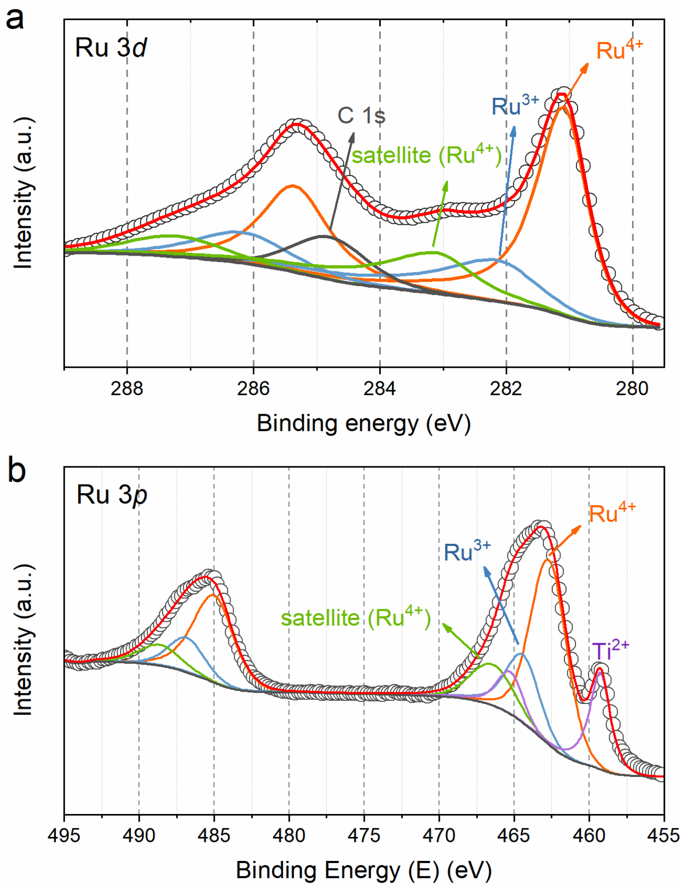

Supplementary Fig. 33 | XPS spectra of py-RuO_2:Zn catalyst. Core-level (a) Ru 3*d* and (b) Ru 3*p* XPS spectra for the py-RuO_2:Zn catalyst post the OER test at 10 mA cm^{-2} for 350 h in 0.5 M H_2SO_4 solution.

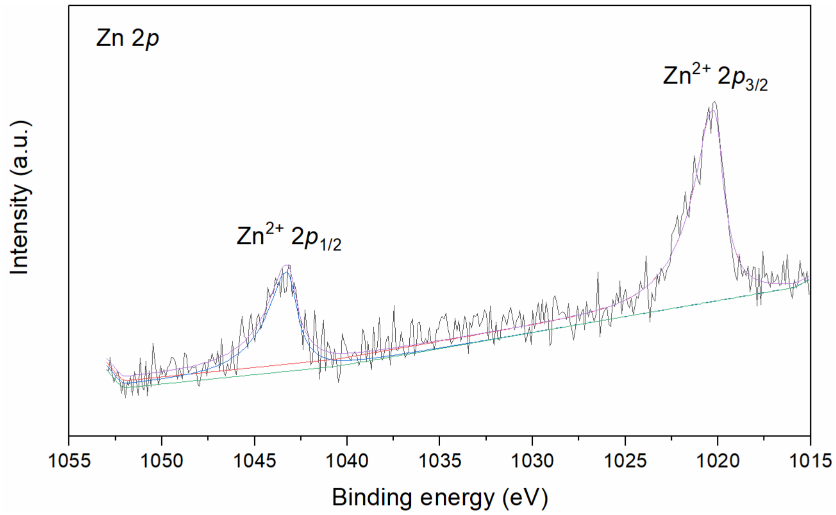

Supplementary Fig. 34 | Core-level Zn 2*p* XPS spectra for the py-RuO_2:Zn catalyst post the OER test at 10 mA cm^{-2} for 350 h in 0.5 M H_2SO_4 solution.

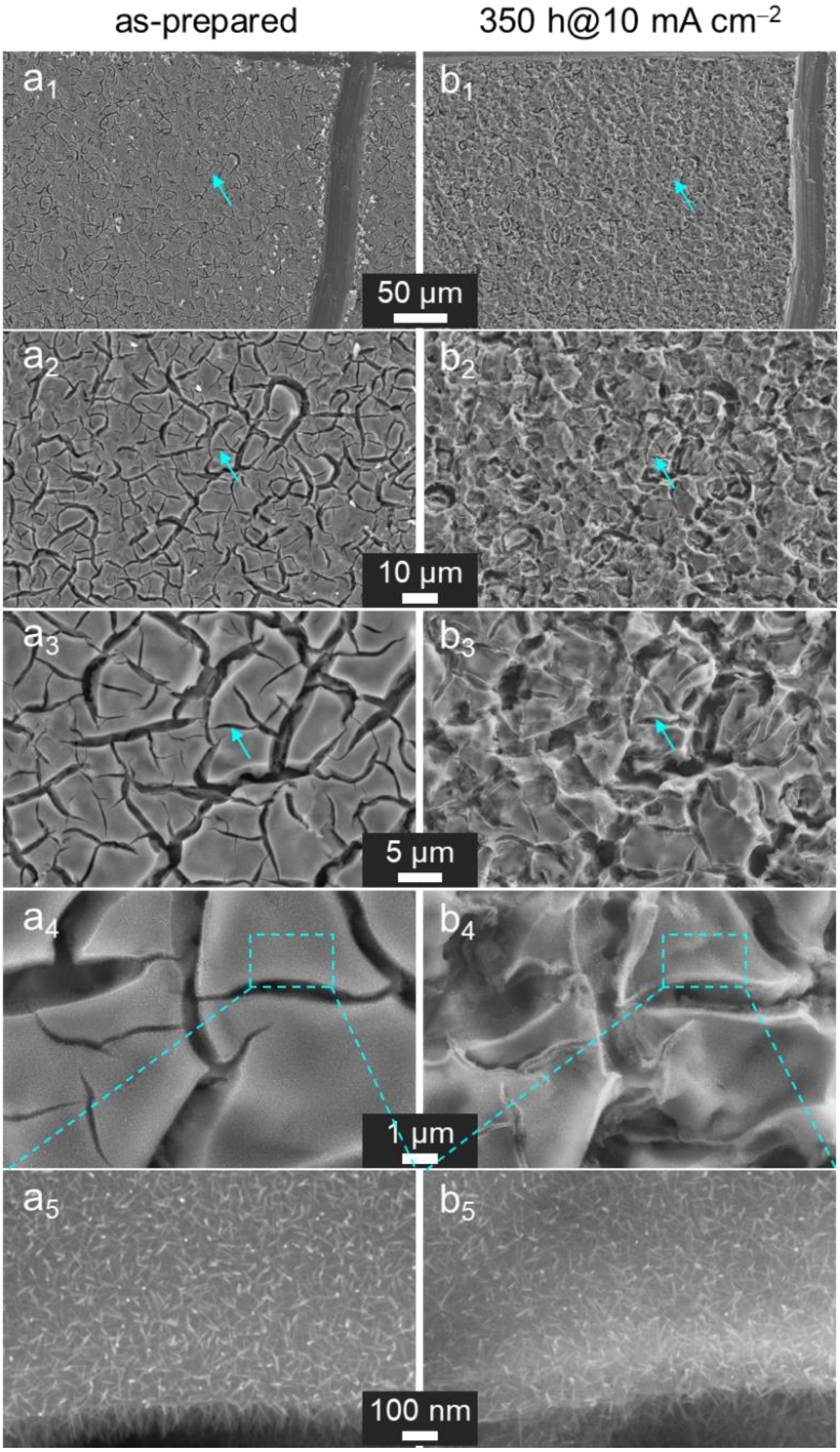

Supplementary Fig. 35 | Quasi-in situ SEM images of py-RuO_2:Zn catalyst. (a_1–a_5) before and (b_1–b_5) after an OER stability test at 10 mA cm^{-2} for 350 h.

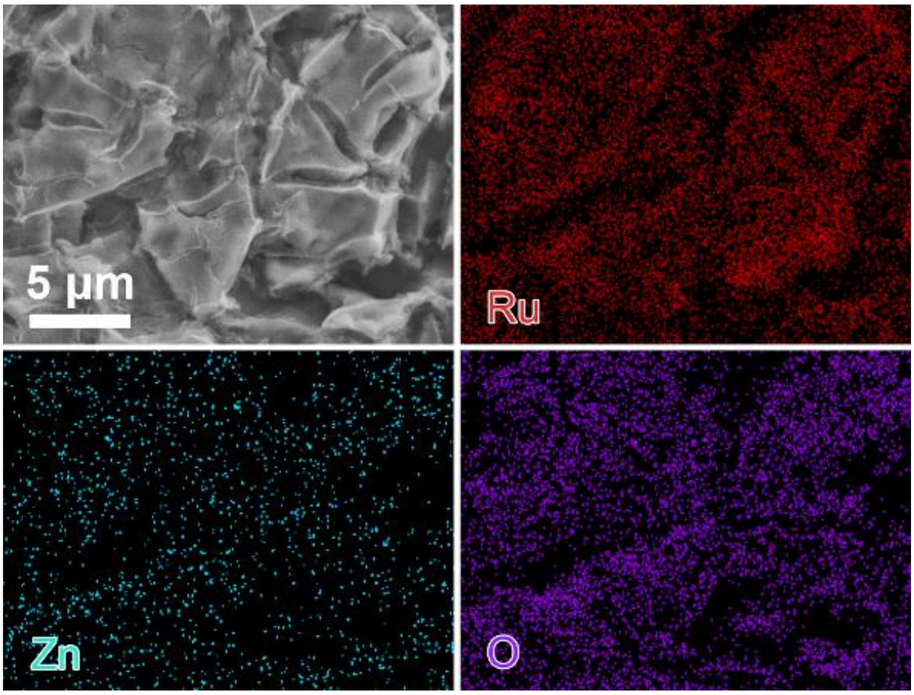

Supplementary Fig. 36 | SEM image and EDS analysis of py-RuO_2:Zn catalyst after an OER stability test at 10 mA cm^{-2} for 350 h.

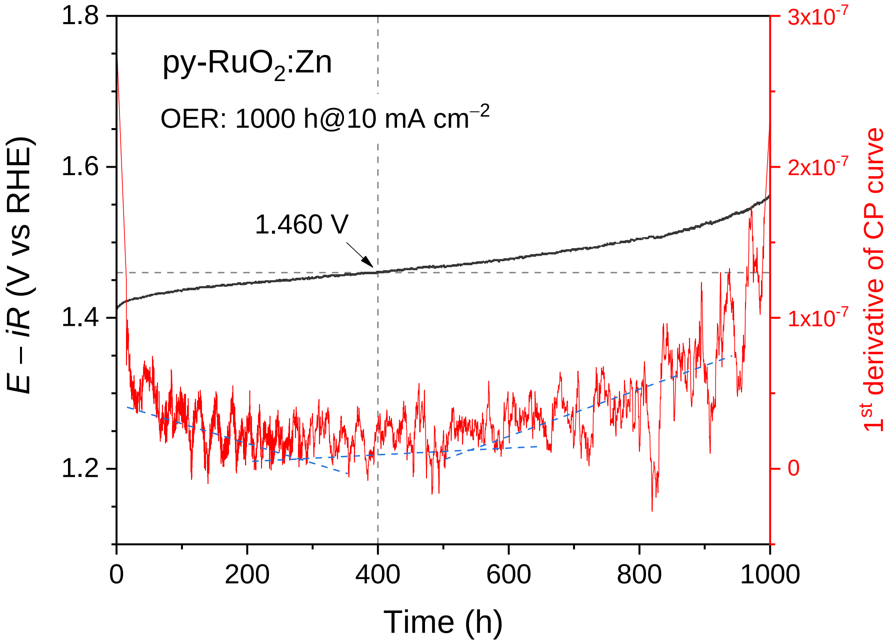

Supplementary Fig. 37 | CP curve and its 1st derivative curve for OER stability test. The OER stability of the py-RuO_2:Zn catalyst was measured at 10 mA cm^{-2} for 1000 h in 0.5 M H_2SO_4. The value of 1.460 V is to guide the approximate inflection region on the curve.

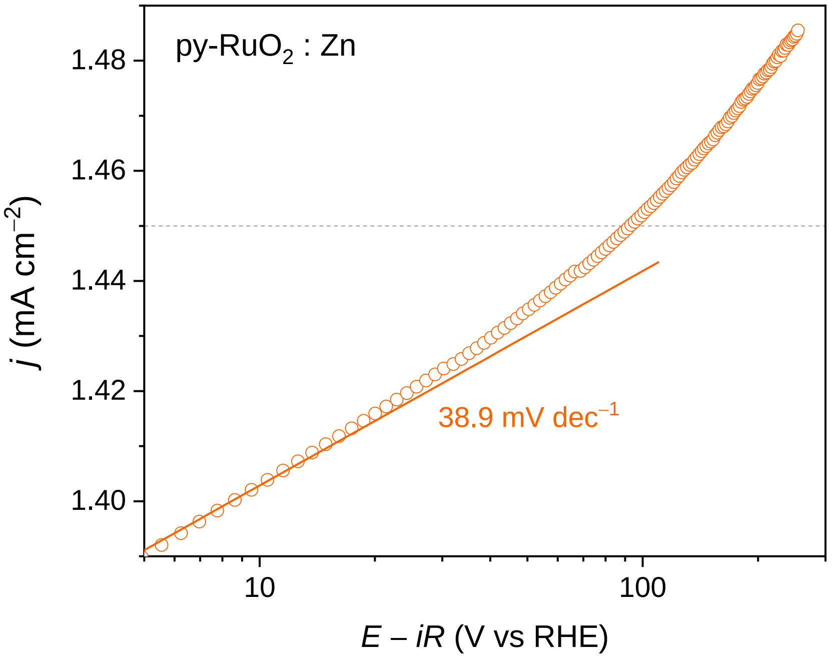

Supplementary Fig. 38 | Tafel slope for OER on the py-RuO_2:Zn catalyst in O_2-saturated 0.5 M H_2SO_4 solution.

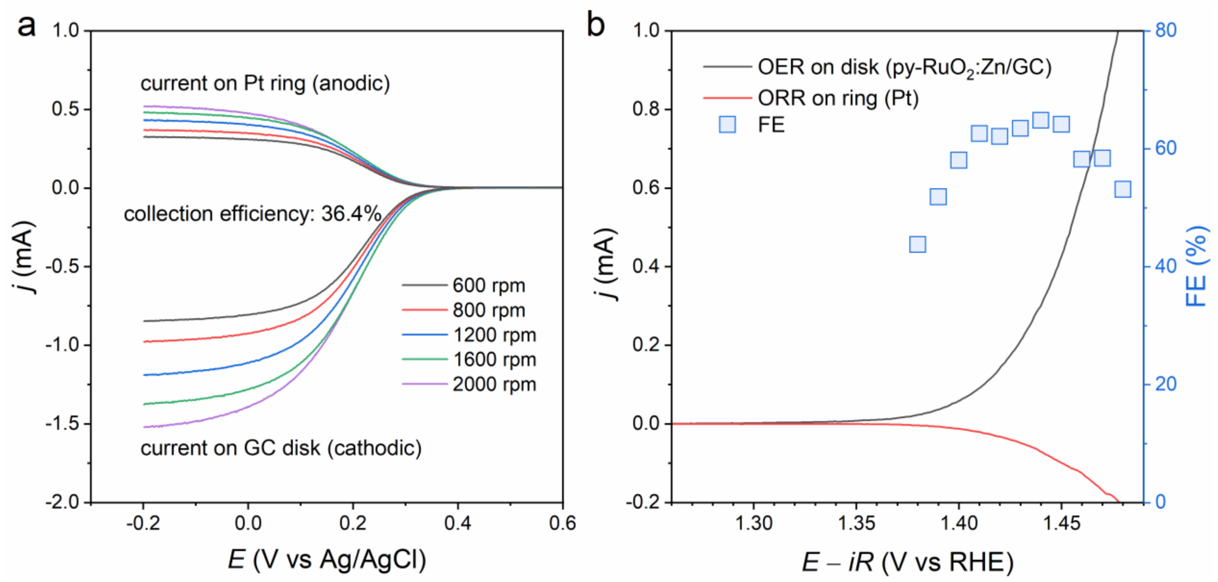

Supplementary Fig. 39 | OER process investigations at low overpotentials using a rotating ring-disk (Pt-GC) electrode (RRDE). (a) Calibration of the collection efficiency of RRDE using the $Fe(CN)_6^{3-}/Fe(CN)_6^{4-}$ redox couple in Ar-saturated solution of 0.1 M KNO_3 and 10 mM $K_3Fe(CN)_6$. Pt ring potential was held at 0.8 V vs Ag/AgCl. (b) Disk and ring currents collected in the RRDE measurement for OER on the py-RuO_2:Zn catalyst in Ar-saturated solution of 0.5 M H_2SO_4, together with the calculated faradaic efficiency (FE) of OER. The catalyst with a loading of 15 μg cm^{-2} was dispersed on the GC disk electrode. Pt ring potential was held at 0.1 V vs RHE, while a potential scan rate of 2 mV s^{-1} was performed on the disk electrode. The RRDE was operated at a constant rotation rate of 1600 rpm. The corresponding faradaic efficiency of OER measured by RRDE can be calculated as $FE_{OER} = i_{ring}/(i_{disk} \times N)$, where i_{ring} and i_{disk} are the measured current on disk and ring electrodes, respectively, and N is the collection efficiency of RRDE, assuming only a 4e^- ORR process existing on the Pt ring electrode.

In this work, an overpotential of 173 mV at 10 mA cm^{-2} was observed on the LSV curve of OER for the Zn-doped RuO_2 (py-RuO_2:Zn) catalyst, which was about 200 mV lower than that for the commercial RuO_2 catalyst. We then studied the electrode process at low potentials around 1.40 V_{RHE} using a rotating ring-disk (Pt-GC) electrode (RRDE, E7R9PTGC from PINE Research Instrum.) setup to understand whether it was the 4e^- oxidation of water to O_2. Collection efficiency of the RRDE (N) was first calibrated with the $Fe(CN)_6^{3-}/Fe(CN)_6^{4-}$ redox couple (Supplementary Fig. 39a). The measured value was $N = 36.4\%$, well agreeing with the parameter, 37%, provided by the manufacturer. RRDE measurement of OER was then performed on the py-RuO_2:Zn catalyst with a reduced loading of 15 μg cm^{-2} to minimize the disturbance from O_2 bubble accumulation.[3] As shown in Supplementary Fig. 39b, anodic current appeared on the catalyst loaded GC disk

electrode when the potential swept to about 1.35 V. Meanwhile, clear cathodic current was observed on the Pt ring, indicating an explicit reduction of the products released from the disk electrode. The products could be the O_2, dissolved Ru cations, and/or H_2O_2. In the stability test at 10 mA cm^{-2} we have found a good stability number (molar ratio of the evolved O_2 to the dissolved Ru element) of the py-RuO_2:Zn catalyst (Fig. 3e, lower plot in the main text). Thus, the contribution of Ru dissolution/reduction to the anodic/cathodic current on the disk/ring electrodes would be negligible. For the possible production of H_2O_2, Nørskov and colleagues recently reported a $2e^-$ oxidation path of water to H_2O_2 in addition to the $4e^-$ path to O_2 (i.e., OER). However, they also found that the OER path was thermodynamically more favorable on RuO_2,[4] consistent with the previous research from Dousikou and co-authors who found an electrooxidation of H_2O_2 on RuO_2 beginning at potentials around 1.0 V, negative to the standard thermodynamic potential of OER.[5] Therefore, it seems unlikely that the $2e^-$ oxidation of water to H_2O_2 has contributed to the observed current on the disk.

We further calculated the corresponding faradaic efficiency of OER ($FE_{OER} = i_{ring}/(i_{disk} \times N)$, assuming only a $4e^-$ ORR process existing on the Pt ring electrode). As shown in Supplementary Fig. 40b, a volcanic trend in the FE_{OER} was found with a maximum value of about 65%. Obviously, the measured FE_{OER} were generally lower than the expected FE_{OER} = 100%. The deviation probably stems from two sources: (i) the low efficient mass transfer of the produced O_2 from the ring to the disk, attributed to the slow kinetics of O_2 gas dissolution; (ii) the reduced Pt ring activity for ORR, due to the deposition of dissolved Ru on the Pt surface. During the RRDE test, a part of the O_2 product may accumulate on the surface of catalyst due to the slow kinetics of O_2 gas dissolution, thereby reducing the amount of dissolved O_2 that can transfer to the ring electrode and the related FE_{OER}. But with the potential increasing more O_2 was produced, alleviating the impact of O_2 accumulation. Thus, there was a gradual increase of FE_{OER} (the left branch of the FE_{OER} plot). However, at more positive potentials the formation and release of O_2 bubble was accelerated. As a competitive process of oxygen dissolution, it would inevitably impair the measurement of FE_{OER} (the right branch of the FE_{OER} plot).[3] In addition, the dissolved Ru from the catalyst (disk) can be also reduced on the Pt ring electrode. It was reported that the deposition of Ru has detrimental impact on the ORR activity of the Pt ring electrode, although the amount of Ru was small.[6] In short, the results of RRDE more support the observed anodic process as the $4e^-$ oxidation of water to oxygen, *i.e.*, OER, rather than other water oxidation processes.

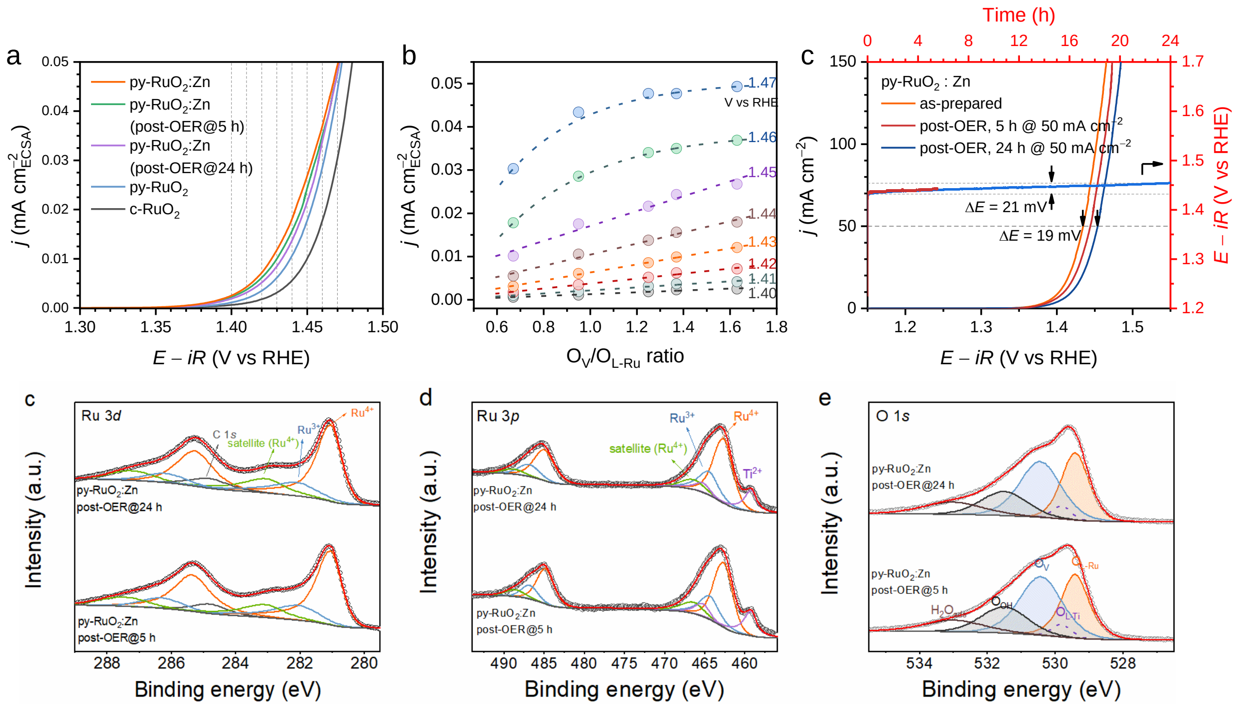

Supplementary Fig. 40 | OER performance characterization and XPS analysis of catalysts. (a) ECSA normalized LSV curves for OER on py-RuO_2:Zn, py-RuO_2, and c-RuO_2 catalysts, together with two py-RuO_2:Zn samples that have been aged at 50 mA cm^{-2} for 5 h and 24 h. (b) The dependence of specific OER current density on the concentration of V_O defects ($O_V/O_{L\text{-}Ru}$ ratio) at given electrode potentials. (c) LSV and CP curves of as-prepared py-RuO_2:Zn catalyst following acidic OER tests for 5 h and 24 h at 50 mA cm^{-2}. Core-level (d) Ru 3*d*, (e) Ru 3*p*, and (f) O 1*s* XPS spectra on the py-RuO_2:Zn catalysts post OER test at 50 mA cm^{-2} for 5 h and 24 h in 0.5 M H_2SO_4 solution.

To experimentally validate the key role of V_O defects in OER activity, specific current densities determined from ECSA-normalized LSV curves were plotted against the V_O concentrations ($O_V/O_{L\text{-}Ru}$ ratio) measured by XPS. Several further reference samples were prepared, py-RuO_2:Zn (post-OER@5 h) and py-RuO_2:Zn (post-OER@24 h), corresponding to the py-RuO_2:Zn catalysts after OER for 5 h and 24 h at a high current density of 50 mA cm^{-2}, respectively (Supplementary Fig. 40c). XPS results reveal a slight decrease in the concentrations of V_O defects and Ru^{3+} species on the aged samples. The ECSA normalized LSV curves are displayed in Supplementary Fig. 40a, from which the specific current densities at different potentials were obtained and plotted in Supplementary Fig. 40b. Obviously, there is strong dependence of the current density on the V_O defect concentration, with a good linear relationship established at potentials lower than 1.45 V.

Below 1.45 V, an increase of V_O defect concentration resulted in enhanced OER activity. When reaction potential was increased to 1.46 V or higher, the linear relationship failed, with the OER activity approaching a limit as the concentration of V_O defects increased. We further noticed that the value of 1.46 V is identical to the threshold potential (~1.46 V) for the rapid dissolution of Ru from the py-RuO_2:Zn catalyst. Hence, the increased Ru dissolution at potentials of 1.46 V and above breaks the linear relationship between OER activity and V_O defect concentration. These observations allow us to conclude that V_O defects play a critical role in catalyzing OER on the py-RuO_2:Zn catalyst.

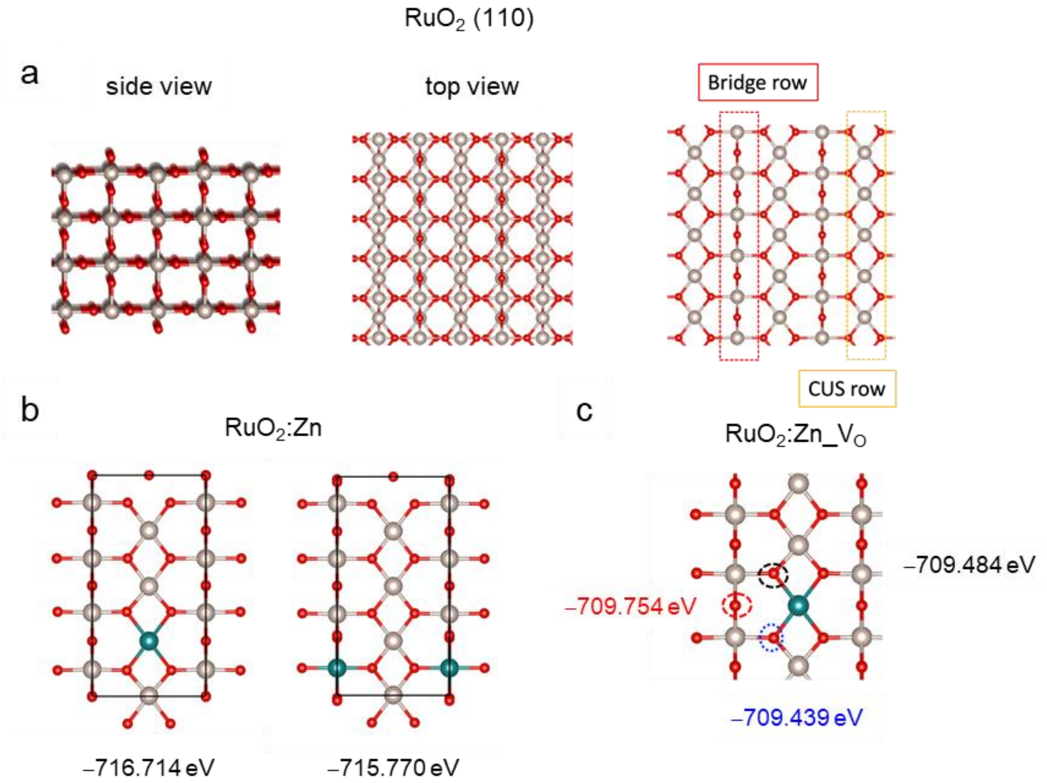

Supplementary Fig. 41 | Optimized structures of catalysts. (a) pristine RuO_2 (110) surface (side view, top view, and Ru sites with different coordination), (b) Zn doped RuO_2 (RuO_2:Zn) surface, and V_O-containing RuO_2:Zn (RuO_2:Zn_V_O) surface.

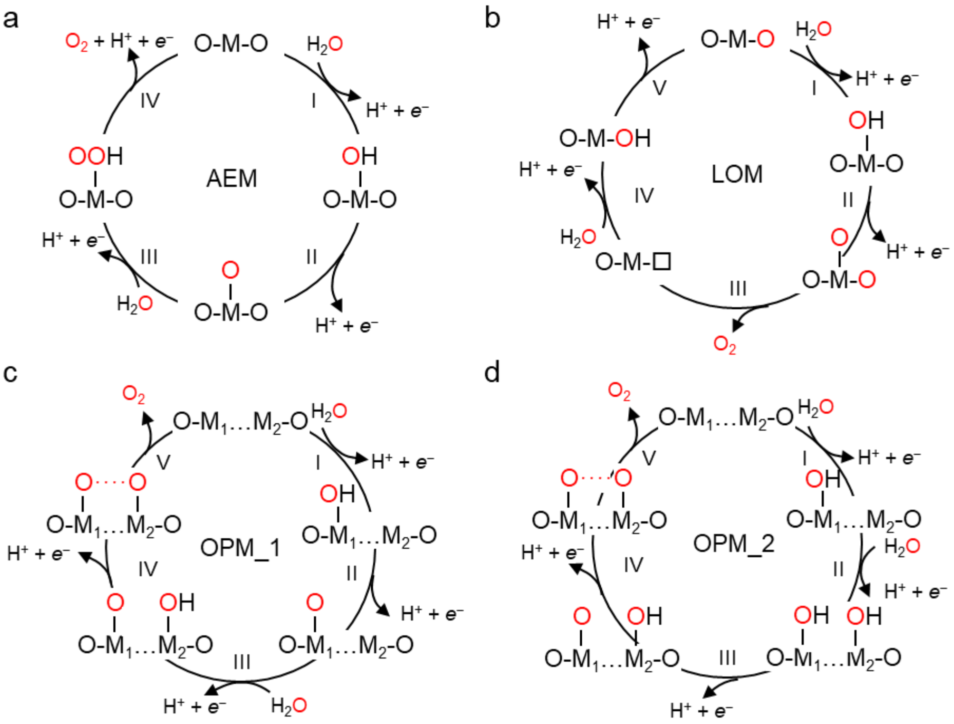

Supplementary Fig. 42 | OER mechanisms used for theoretic calculation. The different reaction pathways of OER proceeds with the four electron-proton transfers were considered as:

The adsorbate evolution mechanism (AEM):[7]

$H_2O_{(l)} + * \rightarrow *OH + H^+ + e^-$ (4)

$*OH \rightarrow *O + H^+ + e^-$ (5)

$*O + H_2O \rightarrow *OOH + H^+ + e^-$ (6)

$*OOH \rightarrow O_2 + H^+ + e^-$ (7)

$$\Delta G_1 = \Delta G_{\mathrm{OH}} \quad (8)$$

$$\Delta G_2 = \Delta G_{\mathrm{O}} - \Delta G_{\mathrm{OH}} \quad (9)$$

$$\Delta G_3 = \Delta G_{\mathrm{OOH}} - \Delta G_{\mathrm{O}} \quad (10)$$

$$\Delta G_4 = 4.92 - \Delta G_{\mathrm{OOH}} \quad (11)$$

$$\Delta G_{\mathrm{max}} = \max(\Delta G_1, \Delta G_2, \Delta G_3, \Delta G_4) \quad (12)$$

$$\Delta G_{\mathrm{OH}} = E_{\mathrm{slab,OH}} - E_{\mathrm{slab}} - (E_{\mathrm{H_2O}} - \tfrac{1}{2}E_{\mathrm{H_2}}) \quad (13)$$

$$\Delta G_{\mathrm{O}} = E_{\mathrm{slab,O}} - E_{\mathrm{slab}} - (E_{\mathrm{H_2O}} - E_{\mathrm{H_2}}) \quad (14)$$

$$\Delta G_{\mathrm{OOH}} = E_{\mathrm{slab,OOH}} - E_{\mathrm{slab}} - (2E_{\mathrm{H_2O}} - \tfrac{3}{2}E_{\mathrm{H_2}}) \quad (15)$$

The free energy of H_2O was computed at 0.035 bar and 298 K.

The lattice oxygen mechanism (LOM):[7]

$H_2O_{(l)} + * \rightarrow *OH + H^+ + e^-$ (16)

$*OH \rightarrow *O + H^+ + e^-$ (17)

$*O + M\text{–}O \rightarrow O_2 + *$ (18)

$M\text{–}Voc + H_2O_{(l)} \rightarrow *OH + H^+ + e^-$ (19)

$*OH \rightarrow M\text{–}O + H^+ + e^-$ (20)

Here, the *OH in (16) and (17) have the O as the lattice O.

$$\Delta G_{1'} = \Delta G_{OH} \quad (21)$$

$$\Delta G_{2'} = \Delta G_{O} - \Delta G_{OH} \quad (22)$$

$$\Delta G_{3'} = \Delta G_{OH} - \Delta G_{O} \quad (23)$$

$$\Delta G_{4'} = 4.92 - \Delta G_{OH} \quad (24)$$

$$\Delta G_{max} = \max(\Delta G_{1'}, \Delta G_{2'}, \Delta G_{3'}, \Delta G_{4'}) \quad (25)$$

$$\Delta G_{OH} = E_{slab,OH} - E_{slab} - (E_{H_2O} - \frac{1}{2} E_{H_2}) \quad (26)$$

$$\Delta G_{O} = E_{slab,O} - E_{slab} - (E_{H_2O} - E_{H_2}) \quad (27)$$

$$\Delta G_{OH} = E_{slab,OH} - E_{slab} + E_{O_2} - (2E_{H_2O} - \frac{3}{2} E_{H_2}) \quad (28)$$

The free energy of O_2 was computed from the reaction $H_2O \rightarrow H_2 + O_2$

The dual site mechanism, also known as oxide path mechanism (OPM):[8]

OPM_1:

$H_2O_{(l)} + * \rightarrow *OH + H^+ + e^-$ (29)

$*OH \rightarrow *O + H^+ + e^-$ (30)

$*O + H_2O_{(l)} \rightarrow *O\ldots*OH + H^+ + e^-$ (31)

$*O\ldots*OH \rightarrow *O\ldots*O + H^+ + e^-$ (32)

$*O\ldots*O \rightarrow O_2 + *$ (33)

$$\Delta G_{1''} = \Delta G_{OH} \quad (34)$$

$$\Delta G_{2''} = \Delta G_{O} - \Delta G_{OH} \quad (35)$$

$$\Delta G_{3''} = \Delta G_{O\ldots OH} - \Delta G_{O} \quad (36)$$

$$\Delta G_{4''} = 4.92 - \Delta G_{O\ldots OH} \quad (37)$$

$$\Delta G_{max} = \max(\Delta G_{1''}, \Delta G_{2''}, \Delta G_{3''}, \Delta G_{4''}) \quad (38)$$

$$\Delta G_{OH} = E_{slab,OH} - E_{slab} - (E_{H_2O} - \frac{1}{2} E_{H_2}) \quad (39)$$

$$\Delta G_{O} = E_{slab,O} - E_{slab} - (E_{H_2O} - E_{H_2}) \quad (40)$$

$$\Delta G_{O\ldots OH} = E_{slab,O\ldots OH} - E_{slab} - (2E_{H_2O} - \frac{3}{2} E_{H_2}) \quad (41)$$

OPM_2

$H_2O_{(l)} + * \rightarrow *OH + H^+ + e^-$ (42)

$*OH + H_2O_{(l)} \rightarrow *OH\ldots*OH + H^+ + e^-$ (43)

$*OH\ldots*OH \rightarrow *O\ldots*OH + H^+ + e^-$ (44)

$*O\ldots*OH \rightarrow *O\ldots*O + H^+ + e^-$ (45)

$*O\ldots*O \rightarrow O_2 + *$ (46)

$$\Delta G_{1'''} = \Delta G_{\mathrm{OH}} \quad (47)$$

$$\Delta G_{2'''} = \Delta G_{\mathrm{O}} - \Delta G_{\mathrm{OH}} \quad (48)$$

$$\Delta G_{3'''} = \Delta G_{\mathrm{O\ldots OH}} - \Delta G_{\mathrm{O}} \quad (49)$$

$$\Delta G_{4'''} = 4.92 - \Delta G_{\mathrm{O\ldots OH}} \quad (50)$$

$$\Delta G_{\mathrm{max}} = \max(\Delta G_{1}''', \Delta G_{2}''', \Delta G_{3}''', \Delta G_{4}''') \quad (51)$$

$$\Delta G_{\mathrm{OH}} = E_{\mathrm{slab,OH}} - E_{\mathrm{slab}} - (E_{\mathrm{H_2O}} - \frac{1}{2} E_{\mathrm{H_2}}) \quad (52)$$

$$\Delta G_{\mathrm{OH\ldots OH}} = E_{\mathrm{slab,OH..OH}} - E_{\mathrm{slab}} - (E_{\mathrm{H_2O}} - E_{\mathrm{H_2}}) \quad (53)$$

$$\Delta G_{\mathrm{O\ldots OH}} = E_{\mathrm{slab,O\ldots OH}} - E_{\mathrm{slab}} - (2E_{\mathrm{H_2O}} - \frac{3}{2} E_{\mathrm{H_2}}) \quad (54)$$

the over potential is computed as:

$$\eta = \frac{\Delta G_{\mathrm{max}}}{e} - 1.23\ \ V \quad (55)$$

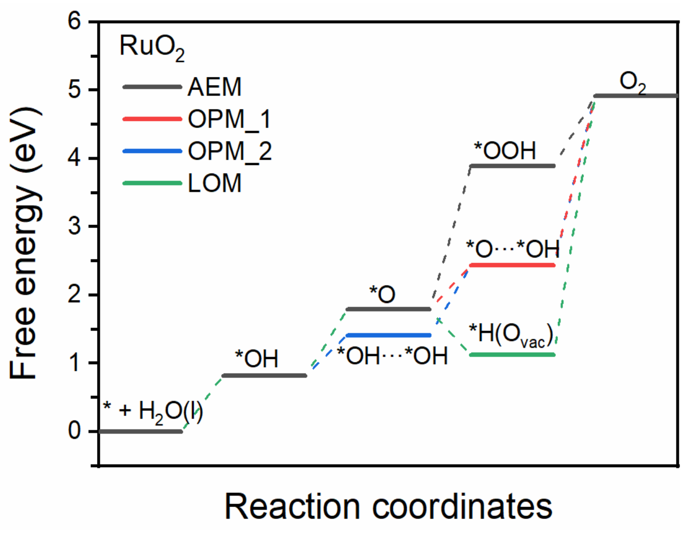

Supplementary Fig. 43 | Calculated free-energy diagrams of different OER paths on the RuO_2 surface.

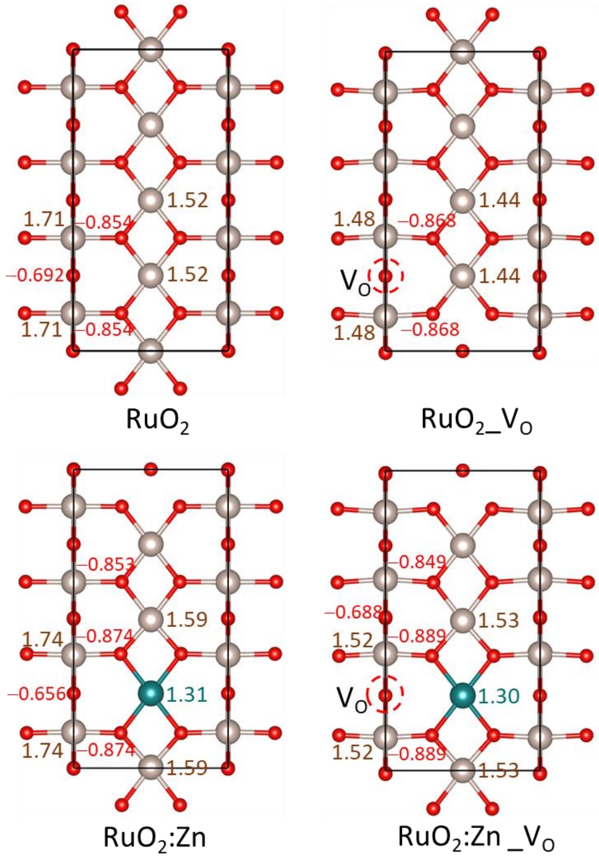

Supplementary Fig. 44 | Bader charge analysis of Ru (brown), Zn (dark cyan), and oxygen (red) sites on the surface of different samples.

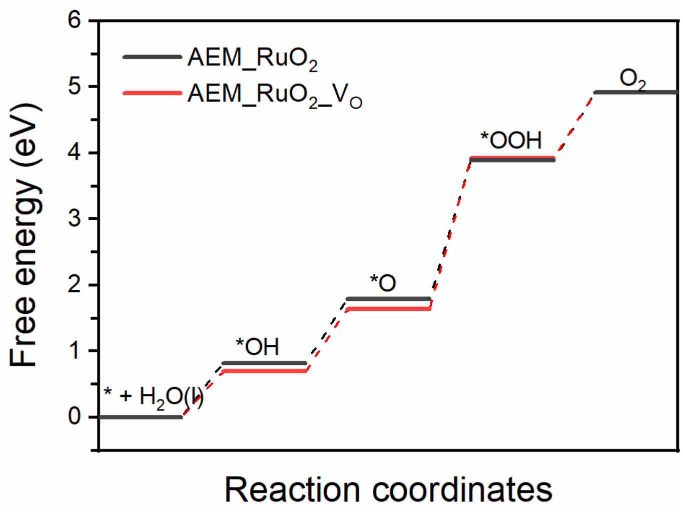

Supplementary Fig. 45 | Calculated free-energy diagrams of AEM paths on the RuO_2 and RuO_2_V_O surface.

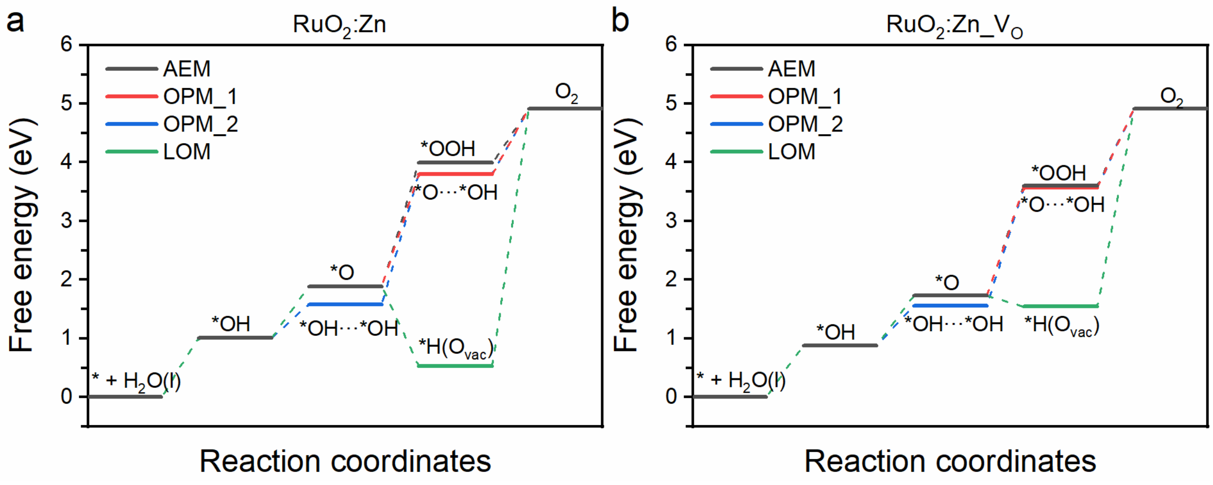

Supplementary Fig. 46 | Calculated free-energy diagrams of different OER paths on different catalyts. (a) RuO_2:Zn and (b) RuO_2:Zn _V_O surfaces.

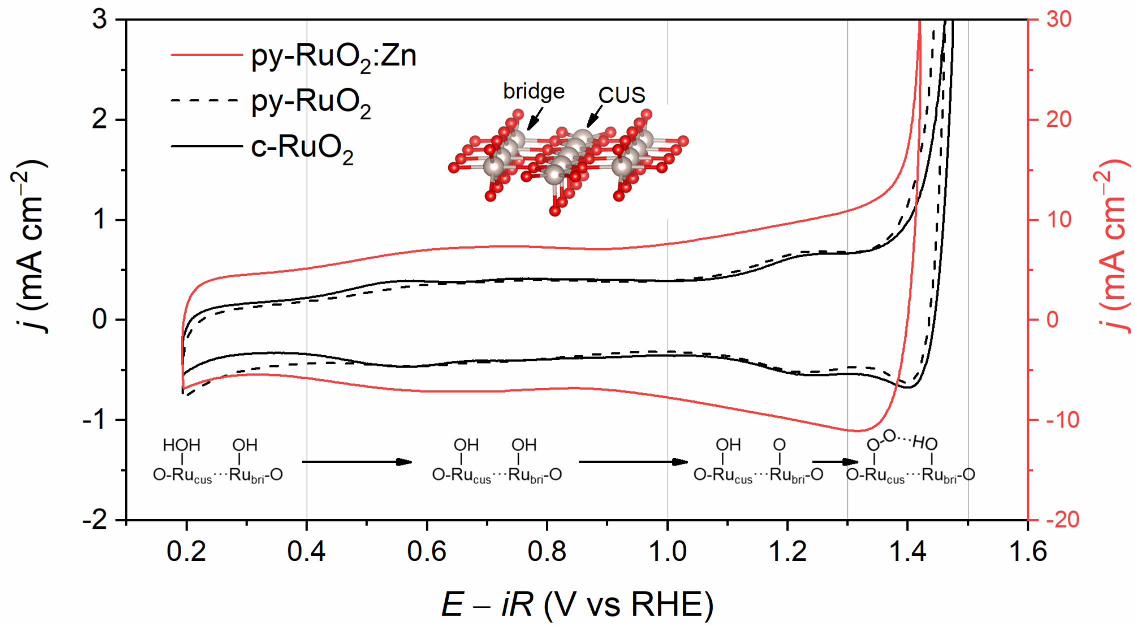

Supplementary Fig. 47 | CV curves of py-RuO_2:Zn, py-RuO_2, and c-RuO_2 catalysts in 0.5 M H_2SO_4 solution. Potential scan rate: 50 mV s^{-1}.

Supplementary Table 1 | The contents of Ru and Zn in the catalysts determined by ICP-MS.[a]

py-RuO_2 : Zn	Ru /μg	Zn /μg	Zn/Ru atomic ratio		
			ICP-MS	EDS (SEM)	EDS (HRTEM)[c]
before acid etching	289.30 (303.21)[b]	102.11 (98.07)[b]	0.546 (0.5)[b]	0.566	—
after acid etching	260.01	10.75	0.0639	0.0515	0.0546

[a]The average value of 5 parallel samples. The mass values reported in this table are based on a geometric area of 0.5 cm^2 for the catalyst coating on the Ti plate.

[b]The values in parentheses are calculated based on the molar amount of precursors.

[c]The value was obtained on a single nanowire measured by EDS during the HADDF-STEM analyses.

Supplementary Table 2 | Results of deconvolution of the Ru $3p_{3/2}$ and O 1*s* XPS spectra of different catalysts.

	Ru $3p_{3/2}$			O 1*s*		
	BE/eV	Proportion[a] (to Ru^{4+})	FWHM	BE/eV	Proportion[a] (to O_{L-Ru})	FWHM
py-RuO_2:Zn	462.82 (Ru^{4+})	1.00	2.70	529.42 (O_{L-Ru})	1.00	0.86
	464.53 (Ru^{3+})	0.55	2.68	530.45 (O_V)	1.63	1.50
	466.45 ($Sat._{Ru4+}$)	0.25	3.20	531.50 (O_{OH})	0.72	1.70
py-RuO_2	462.70 (Ru^{4+})	1.00	2.68	529.42 (O_{L-Ru})	1.00	0.86
	464.50 (Ru^{3+})	0.31	2.65	530.48 (O_V)	0.95	1.50
	466.47 ($Sat._{Ru4+}$)	0.26	3.22	531.49 (O_{OH})	0.40	1.68
c-RuO_2	462.45 (Ru^{4+})	1.00	2.75	529.42 (O_{L-Ru})	1.00	0.85
	464.32 (Ru^{3+})	0.25	2.72	530.47 (O_V)	0.67	1.53
	466.31 ($Sat._{Ru4+}$)	0.20	3.35	531.49 (O_{OH})	0.28	1.68
py-RuO_2:Zn[b]	462.78 (Ru^{4+})	1.00	2.75	529.42 (O_{L-Ru})	1.00	0.98
(post OER)	464.55 (Ru^{3+})	0.40	2.73	530.43 (O_V)	1.18	1.48
	466.57 ($Sat._{Ru4+}$)	0.25	3.30	531.47 (O_{OH})	0.62	1.67
py-RuO_2:Zn[c]	462.80 (Ru^{4+})	1.00	2.75	529.42 (O_{L-Ru})	1.00	0.96
(post-OER@5 h)	464.57 (Ru^{3+})	0.46	2.70	530.44 (O_V)	1.36	1.53
	466.58 ($Sat._{Ru4+}$)	0.24	3.30	531.51 (O_{OH})	0.66	1.70
py-RuO_2:Zn[c]	462.80 (Ru^{4+})	1.00	2.75	529.42 (O_{L-Ru})	1.00	0.95
(post-OER@24 h)	464.56 (Ru^{3+})	0.43	2.68	530.45 (O_V)	1.25	1.50
	466.57 ($Sat._{Ru4+}$)	0.20	3.30	531.51 (O_{OH})	0.60	1.70

[a]The proportion is calculated based on the peak area of the Ru^{3+} and Ru^{4+} species.

[b]After a stability test of OER at 10 mA cm^{-2} for 350 h.

[c]After the stability test of OER at 50 mA cm^{-2} for different time.

Supplementary Table 3 | Summary of the impedance fitting data for py-RuO_2:Zn and py-RuO_2 catalysts in O_2-saturated 0.5 M H_2SO_4.

Potential	R_s (Ω)		R_f (Ω)		CPE_f (mF)		R_{ct} (Ω)		CPE_{ct} (mF)	
(V vs RHE)	py-RuO_2:Zn	py-RuO_2	py-RuO_2:Zn	py-RuO_2	py-RuO_2:Zn	py-RuO_2	py-RuO_2:Zn	py-RuO_2	py-RuO_2:Zn	py-RuO_2
1.20	3.672	3.209	1.346	5.163	286.69	61.91	24358	53538	161.60	14.47
1.30	3.696	3.226	1.231	5.238	284.55	58.82	4148	36207	179.84	15.72
1.325	3.690	3.211	1.219	5.723	286.36	59.15	1751	15826	186.01	15.51
1.35	3.731	3.216	1.031	4.609	251.23	61.01	296.6	2576	195.43	16.13
1.375	3.762	3.241	0.997	3.109	252.84	56.29	43.15	519.2	210.17	17.42
1.40	3.760	3.244	1.101	2.685	298.26	66.52	9.047	114.7	238.01	20.58
1.42	4.040	3.216	1.268	2.403	321.89	95.44	2.930	42.4	301.09	24.30
1.44	4.045	3.210	0.997	2.188	245.43	107.34	1.813	22.91	314.29	27.68
1.46	4.071	3.201	0.802	1.781	218.90	189.30	1.296	11.04	312.64	29.85

Supplementary Table 4 | Summary of recently reported Ru-based catalysts for acidic water oxidation.

Catalysts	η_{10} (mV)	Tafel slope (mV dec^{-1})	Mass activity@η (mA mg^{-1}@mV)	Stability under 10 mA cm^{-2} (ΔE@duration time)	Electrolytes	References
py-RuO_2:Zn nanowires array	173	41.2	881@300	60 mV@500 h<br>153 mV@1000 h	0.5 M H_2SO_4	This work
$Bi_{1.5}Er_{0.5}Ru_2O_7$	180	51.3	–	–@100 h	0.1 M $HClO_4$	Nat. Commun. **2022**, *13*, 4106[9]
C-RuO_2-RuSe	212	49.5	–	–@50 h	0.5 M H_2SO_4	Chem **2022**, *8*, 1673–1687[10]
RuO_2-WC NPs	347	88.5	1430@320	–@10 h	0.5 M H_2SO_4	Angew. Chem. Int. Ed. **2022**, *61*, e202202519[11]
$Mn_{0.73}Ru_{0.27}O_{2-\delta}$	208	65.3	879.2@270	–@10 h	0.5 M H_2SO_4	Energy Environ. Sci. **2022**, *15*, 2356–2365[12]
Pt-RuO_2	228	51	–	–@100 h	0.5 M H_2SO_4	Sci. Adv. **2022**, *8*, eabl9271[13]
Li_xRuO_2	156	83.3	~253@300	120 mV@70 h	0.5 M H_2SO_4	Nat. Commun. **2022**, *13*, 3784[14]
S_H-RuCuO NRs	231	39.7	783@270	44 mV@250 h	0.5 M H_2SO_4	Nano Res. **2022**, *15*, 3964–3970[15]
RuMn	~240	–	–	100 mV@720 h	0.5 M H_2SO_4	Adv. Funct. Mater. **2022**, *32*, 2200131[16]
RuO_2/D-TiO_2	180	43	–	–@100 h	0.5 M H_2SO_4	ACS Catal. **2022**. *12*, 9437–9445[17]
$RuPbO_x$	191	39	593@270	85 mV@100 h	0.5 M H_2SO_4	Chin. J. Catal. **2022**, *43*, 130–138[18]
E-Zn-RuO_2	190	50.9	–	–@60 h	0.5 M H_2SO_4	J. Mater. Chem. A **2022**, *10*, 16193–16203[19]
$In_{0.17}Ru_{0.83}O_2$	177	32.6	1094.9@300	–@20 h	0.5 M H_2SO_4	J. Mater. Chem. A **2022**, *10*, 3722–3731[20]
RuMn NSBs	196	47.3	–	–@140 h	0.5 M H_2SO_4	Adv. Mater. **2021**, *33*, 2105308[21]
Ru/α-MnO_2	161	29.4	~431@161	169 mV@200 h	0.1 M $HClO_4$	Nat. Catal. **2021**, *4*, 1012-1023[3]
Na-a/c-RuO_2	205	48.6	~100@250	–@60 h	0.1 M $HClO_4$	Angew. Chem. Int. Ed. **2021**, *60*, 18821–18829[22]
Ru/RuS_2	201	47.2	~94@300	–@24(i-t) h	0.5 M H_2SO_4	Angew. Chem. Int. Ed. **2021**, *60*, 12328–12334[23]
SrRuIr oxide	190	39	281@270<br>429@300	43 mV@1500 h	0.5 M H_2SO_4	J. Am. Chem. Soc. **2021**, *143*, 6482–6490[24]
Ni-Ru@RuO_x	184	44	315@220	–@30 h	0.5 M H_2SO_4	Adv. *Energy* Mater. **2021**, *11*, 2003448[25]
$Y_{1.7}Sr_{0.3}Ru_2O_7$	264	44.8	418@270<br>1018@300	–@28 h	0.5 M H_2SO_4	ACS Nano **2021**, *15*, 8537–8548[26]
RuIr@CoNC	223	45	2041@300	–@40 h	0.5 M H_2SO_4	ACS Catal. **2021**, *11*, 3402–3413[27]
RuO_2 nanowires	224	48	–	–@12 h	0.1 M $HClO_4$	Adv. Funct. Mater. **2021**, *31*, 2007344[28]
S-$RuFeO_x$	187	40	1180@190	50 mV@1 h	0.1 M $HClO_4$	Adv. Funct. Mater. **2021**,

						2101405[29]
Zn-doped RuO_2	206	45.6	–	–@30 h	0.5 M H_2SO_4	ChemNanoMat **2021**, *7*, 117–121[30]
Ru/Fe oxide nanoassemblies	238	44.8	–	–@9 h	0.5 M H_2SO_4	Nano Energy **2021**, *84*, 105909[31]
defect-rich RuO_2 nanosheets	199	38.2	520@230	–@6 h	0.5 M H_2SO_4	Energy Environ. Sci. **2020**, *13*, 5143–5151[32]
$Y_{1.8}Cu_{0.2}Ru_2O_{7-\delta}$	~258	52	–	–	0.5 M H_2SO_4	J. Am. Chem. Soc. **2020**, *142*, 7883–7888[33]
$W_{0.2}Er_{0.1}Ru_{0.7}O_{2-\delta}$	168	66.8	1518.6@275	83 mV@500 h	0.5 M H_2SO_4	Nat. Commun. **2020**, *11*, 5368[34]
$RuNi_2$@G-250	227	65	57.6@250	–@24 h	0.5 M H_2SO_4	Adv. Mater. **2020**, *32*, 1908126[35]
RuB_2	223	39.8	~100@250	38 mV@10 h	0.5 M H_2SO_4	ACS Energy Lett. **2020**, *5*, 2909–2915[36]
Mn-RuO_2	158	42.94	596.38@270	370 mV@10 h	0.5 M H_2SO_4	ACS Catal. **2020**, *10*, 1152–1160[37]
IrRu@Te	220	35	590@250	–@20 h	0.5 M H_2SO_4	ACS Catal. **2020**, *10*, 3571–3579[38]
Ru nanoparticles	202	69.6	~300@300	–@10 h	0.5 M H_2SO_4	ACS Catal. **2020**, *10*, 12575–12581[39]
Ru_1–Pt_3Cu	220	~51.7	6615@280	–@29 h	0.1 M $HClO_4$	Nat. Catal. **2019**, *2*, 304–313[40]
RuCu nanosheets	236	41	–	–@12 h	0.5 M H_2SO_4	Angew. Chem. Int. Ed. **2019**, *58*, 13983–13988[41]
Co-RuIr	235	66.9	–	–@25 h	0.1 M $HClO_4$	Adv. Mater., **2019**, *31*, 1900510[42]
ultrafine defective RuO_2	179	36.9	–	19 mV@20 h	0.5 M H_2SO_4	Adv. Energy Mater. **2019**, *9*, 1901313[43]
Ruthenate nanosheets	255	38	42@250	260 mV@6 h	0.1 M $HClO_4$	Adv. Energy Mater. **2019**, *9*, 1803795[44]
$Cr_{0.6}Ru_{0.4}O_2$	178	58	229@270	–@10 h	0.5 M H_2SO_4	Nat. Commun. **2019**, *10*, 162[45]
$CaCu_3Ru_4O_{12}$	171	40	1942@270	21 mV@24 h	0.5 M H_2SO_4	Nat. Commun. **2019**, *10*, 3809[46]
Na-doped $SrRuO_3$	170	~40	–	-	0.1 M $HClO_4$	Nat. Commun. **2019**, *10*, 2041[47]
$RuTe_2$ nanorods	245	47	–	–@24 h	0.5 M H_2SO_4	Nat. Commun. **2019**, *10*, 5692[48]
Ru_1-N-C	267	52.6	3540@267	–@30(i-t) h	0.5 M H_2SO_4	Nat. Commun.**2019**, *10*, 4849[49]
NPC@RuO_2	220	68.8	–	–@10 h	0.5 M H_2SO_4	Adv. Funct. Mater. **2019**, *29*, 1901154[50]
Cu-doped RuO_2	188	52	352@350	83 mV@8 h	0.5 M H_2SO_4	Adv. Mater. **2018**, *30*, 1801351[51]
Pd-Ru nanoparticles	225	61	–	–@2 h	0.1 M $HClO_4$	J. Am. Chem. Soc. **2018**, *140*, 12760–12764[52]
Au-Ru nanoparticles	220	62	–	–	0.5 M H_2SO_4	Angew. Chem. Int. Ed. **2018**, *57*, 10241–10245[53]
Ru@IrO_x	282	69.1	644.8@330	–@24(i-t) h	0.5 M H_2SO_4	Chem **2019**, *5*, 445–459[54]
$Y_2[Ru_{1.6}Y_{0.4}]O_{7-\delta}$	~240	37	~480@250	–	0.5 M H_2SO_4	Angew. Chem. Int. Ed. **2018**, *57*, 13877–13881[55]

Supplementary Table 5 | Summary of Ru dissolution rate of RuO_2-based catalysts recently reported for acidic water oxidation.

catalysts	Ru dissolution rate $\mu g\ cm_{geo}^{-2}\ h^{-1}$	Test conditions	References
py-RuO_2:Zn	0.156	at 10 mA cm^{-2} for 500 h in 0.5 M H_2SO_4	This work
commercial RuO_2	~40		
sputtering-prepared crystalline RuO_2	~0.36	at ~1.53 V during potential sweeping with 1 mV s^{-1} in 0.1 M H_2SO_4	Catal. Today 2016, 262, 170–180[56]
Na-a/c-RuO_2	0.383	10 mA cm^{-2} for 24 h in 0.1 M $HClO_4$	Angew. Chem. Int. Ed, 2021, 60, 18821–18829[22]
$Y_2Ru_2O_7$	~0.102	at 1.56 V for 10 h in 0.1 M $HClO_4$	ACS Catal. 2020, 10, 12182–12196[57]
$Gd_2Ru_2O_7$	~0.156		
$Bi_2Ru_2O_7$	~0.168		
$Nd_2Ru_2O_7$	~0.812		
RuO_2-WC	0.516	at 1.55 V for 10 h in 0.5 M H2SO4	Angew. Chem. Int. Ed, 2022, 61, e202202519[11]
Li_xRuO_2	0.229	at 10 mA cm^{-2} for 50 h in 0.5 M H_2SO_4	Nat. Commun. 2022, 13, 3784[14]
$RuNi_2$@G-250	0.326	at 10 mA cm^{-2} for 24 h in 0.5 M H_2SO_4	Adv. Mater. 2020, 32, 1908126[35]

Supplementary Table 6 | Adsorption energies of reaction intermediates.

	ΔOH (eV)	ΔO (eV)	ΔOH…OH for OPM_2 (eV)	ΔOOH (eV)	ΔO…OH for OPM_1 (eV)	ΔH for LOM (eV)	ΔG_{max} (eV)	OP (V)
RuO_2	0.82	1.79	1.41	3.89	2.44	1.13	2.10	0.87
$ZnRuO_2$	1.01	1.89	1.58	3.99	3.80	0.53	1.91	0.68
$ZnRuO_2$_V_O	0.88	1.73	1.55	3.60	3.57	1.54	1.84	0.61
RuO_2_V_O	0.70	1.64		3.92			2.28	1.05

Supplementary Table 7 | Mechanisms of OER process.

AEM	OPM	LOM
$H_2O + * \rightarrow *OH + H^+ + e^-$	$H_2O + * \rightarrow *OH + H^+ + e^-$	$H_2O + * \rightarrow *OH + H^+ + e^-$
$*OH \rightarrow *O + H^+ + e^-$	$*OH \rightarrow *O + H^+ + e^-$	$*OH \rightarrow *O + H^+ + e^-$
$*O + H_2O \rightarrow *OOH + H^+ + e^-$	$*O + H_2O \rightarrow *O…*OH + H^+ + e^-$ …………OPM_1	$*O + M–O \rightarrow O_2 + *$
$*OOH \rightarrow O_2 + H^+ + e^-$	$*OH + H_2O \rightarrow *OH…*OH + H^+ + e^-$	$M–Vac + H_2O \rightarrow *OH + H^+ + e^-$
	$*OH…*OH \rightarrow *O…*OH + H^+ + e^-$ ………OPM_2	$*OH \rightarrow M–O + H^+ + e^-$
	$*O…*OH \rightarrow *O…*O + H^+ + e^-$	
	$*O…*O \rightarrow O_2 + *$	

Supplementary references

1. Jo, H. C.; Kim, K. M.; Cheong, H.; Lee, S.-H.; Deb, S. K. In situ Raman spectroscopy of $RuO_2 \cdot xH_2O$. *Electrochem. Solid-State Lett.* **8**, E39−E41 (2005).
2. Arikawa, T.; Takasu, Y.; Murakami, Y.; Asakura, K.; Iwasawa, Y. Characterization of the structure of RuO_2−IrO_2/Ti electrodes by EXAFS. *J. Phys. Chem. B* **102**, 3736−3741 (1998).
3. Lin, C.; Li, J.-L.; Li, X.; Yang, S.; Luo, W.; Zhang, Y.; Kim, S.-H.; Kim, D.-H.; Shinde, S. S.; Li, Y.-F.; Liu, Z.-P.; Jiang, Z.; Lee, J.-H., In-situ reconstructed Ru atom array on α-MnO_2 with enhanced performance for acidic water oxidation. *Nat. Catal.* **4**, 1012–1023 (2021).
4. Viswanathan, V.; Hansen, H. A.; Nørskov, J. K. Selective electrochemical generation of hydrogen peroxide from water oxidation. *J. Phys. Chem. Lett.* **6**, 4224–4228 (2015).
5. Dousikou, M. F.; Koupparis, M. A.; Efstathiou, C. E. Determination of catalase-like activity in plants based on the amperometric monitoring of hydrogen peroxide consumption using a carbon paste electrode modified with ruthenium(iv) oxide. *Phytochem. Anal.* **17**, 255–261 (2006).
6. Gancs, L.; Hakim, N.; Hult, B.; Mukerjee, S. Dissolution of Ru from PtRu electrocatalysts and its consequences in DMFCS. *ECS Trans.* **3**, 607 (2006).
7. Zagalskaya, A.; Alexandrov, V. Role of defects in the interplay between adsorbate evolving and lattice oxygen mechanisms of the oxygen evolution reaction in RuO_2 and IrO_2. *ACS Catal.* **10**, 3650−3657 (2020).
8. Lin, C.; Li, J.-L.; Li, X.; Yang, S.; Luo, W.; Zhang, Y.; Kim, S.-H.; Kim, D.-H.; Shinde, S. S.; Li, Y.-F.; Liu, Z.-P.; Jiang, Z.; Lee, J.-H. In-situ reconstructed Ru atom array on α-MnO2 with enhanced performance for acidic water oxidation. *Nat. Catal.* **4**, 1012−1023 (2021).
9. Zhou, G.; Wang, P.; Hu, B.; Shen, X.; Liu, C.; Tao, W.; Huang, P.; Liu, L. Spin-related symmetry breaking induced by half-disordered hybridization in $Bi_xEr_{2-x}Ru_2O_7$ pyrochlores for acidic oxygen evolution. *Nat. Commun.* **13**, 4106 (2022).
10. Wang, J.; Cheng, C.; Yuan, Q.; Yang, H.; Meng, F.; Zhang, Q.; Gu, L.; Cao, J.; Li, L.; Haw, S.-C.; Shao, Q.; Zhang, L.; Cheng, T.; Jiao, F.; Huang, X. Exceptionally active and stable RuO_2 with interstitial carbon for water oxidation in acid. *Chem* **8**, 1673–1687 (2022).
11. Sun, S.-C.; Jiang, H.; Chen, Z.-Y.; Chen, Q.; Ma, M.-Y.; Zhen, L.; Song, B.; Xu, C.-Y., Bifunctional WC-supported RuO_2 nanoparticles for robust water splitting in acidic media. *Angew. Chem. Int. Ed.* **61**, e202202519 (2022).
12. Wang, K.; Wang, Y.; Yang, B.; Li, Z.; Qin, X.; Zhang, Q.; Lei, L.; Qiu, M.; Wu, G.; Hou, Y., Highly active ruthenium sites stabilized by modulating electron-feeding for sustainable acidic oxygen-evolution electrocatalysis. *Energy Environ. Sci.* **15**, 2356–2365 (2022).
13. Wang, J.; Yang, H.; Li, F.; Li, L.; Wu, J.; Liu, S.; Cheng, T.; Xu, Y.; Shao, Q.; Huang, X., Single-site Pt-doped RuO_2 hollow nanospheres with interstitial C for high-performance acidic overall water splitting. *Sci. Adv.* **8**, eabl9271 (2022).
14. Qin, Y.; Yu, T.; Deng, S.; Zhou, X.-Y.; Lin, D.; Zhang, Q.; Jin, Z.; Zhang, D.; He, Y.-B.; Qiu, H.-J.; He, L.; Kang, F.; Li, K.; Zhang, T.-Y., RuO_2 electronic structure and lattice strain dual engineering for enhanced acidic oxygen evolution reaction performance. *Nat. Commun.* **13**, 3784 (2022).
15. Yao, Q.; Yu, Z.; Chu, Y.-H.; Lai, Y.-H.; Chan, T.-S.; Xu, Y.; Shao, Q.; Huang, X. S Incorporated RuO_2-based nanorings for active and stable water oxidation in acid. *Nano Res.* **15**, 3964–3970 (2022).
16. An, L.; Yang, F.; Fu, C.; Cai, X.; Shen, S.; Xia, G.; Li, J.; Du, Y.; Luo, L.; Zhang, J. A functionally stable RuMn electrocatalyst for oxygen evolution reaction in acid. *Adv. Funct. Mater.* **32**, 2200131 (2022).
17. Wang, X.; Wan, X.; Qin, X.; Chen, C.; Qian, X.; Guo, Y.; Xu, Q.; Cai, W.-B.; Yang, H.; Jiang, K. Electronic structure modulation of RuO_2 by TiO_2 enriched with oxygen vacancies to boost acidic O_2 evolution. *ACS Catal.* **12**, 9437–9445 (2022).
18. Huang, R.; Wen, Y.; Peng, H.; Zhang, B. Improved kinetics of OER on Ru-Pb binary electrocatalyst by decoupling proton-electron transfer. *Chin. J. Catal.* **43**, 130–138 (2022).
19. Zhou, Y.-N.; Yu, N.; Lv, Q.-X.; Liu, B.; Dong, B.; Chai, Y.-M. Surface evolution of Zn doped-RuO_2 under different etching

methods towards acidic oxygen evolution. *J. Mater. Chem. A* **10**, 16193–16203 (2022).

20. Chen, S.; Wang, C.; Gao, F.; Yang, Y.; Huang, M.; Tong, H.; Cheng, Z.; Wang, P.; Wang, P.; Tu, J.; Zeng, X.; Chen, Q., An indium-induced-synthesis $In_{0.17}Ru_{0.83}O_2$ nanoribbon as highly active electrocatalyst for oxygen evolution in acidic media at high current densities above 400 mA cm^{-2}. *J. Mater. Chem. A* **10**, 3722–3731 (2022).

21. Li, L.; Bu, L.; Huang, B.; Wang, P.; Shen, C.; Bai, S.; Chan, T.-S.; Shao, Q.; Hu, Z.; Huang, X., Compensating electronic effect enables fast site-to-site electron transfer over ultrathin RuMn nanosheet branches toward highly electroactive and stable water splitting. *Adv. Mater.* **33**, 2105308 (2021).

22. Zhang, L.; Zhang, H.; Liu, H.; Kim, M. G.; Yang, D.; Liu, S.; Liu, X.; Cho, J., Sodium-decorated amorphous/crystalline RuO_2 with rich oxygen vacancies: A robust pH-universal oxygen evolution electrocatalyst. *Angew. Chem. Int. Ed.* **60**, 18821–18829 (2021).

23. Zhu, J.; Guo, Y.; Liu, F.; Xu, H.; Gong, L.; Shi, W.; Chen, D.; Wang, P.; Yang, Y.; Zhang, C.; Wu, J.; Luo, J.; Mu, S., Regulative electronic states around ruthenium/ruthenium disulphide heterointerfaces for efficient water splitting in acidic media. *Angew. Chem. Int. Ed.* **60**, 12328–12334 (2021).

24. Wen, Y.; Chen, P.; Wang, L.; Li, S.; Wang, Z.; Abed, J.; Mao, X.; Min, Y.; Dinh, C. T.; Luna, P. D.; Huang, R.; Zhang, L.; Wang, L.; Wang, L.; Nielsen, R. J.; Li, H.; Zhuang, T.; Ke, C.; Voznyy, O.; Hu, Y.; Li, Y.; Goddard Iii, W. A.; Zhang, B.; Peng, H.; Sargent, E. H., Stabilizing highly active Ru sites by suppressing lattice oxygen participation in acidic water oxidation. *J. Am. Chem. Soc.* **143**, 6482–6490 (2021).

25. Harzandi, A. M.; Shadman, S.; Nissimagoudar, A. S.; Kim, D. Y.; Lim, H.-D.; Lee, J. H.; Kim, M. G.; Jeong, H. Y.; Kim, Y.; Kim, K. S., Ruthenium core–shell engineering with nickel single atoms for selective oxygen evolution via nondestructive mechanism. *Adv. Energy Mater.* **11**, 2003448 (2021).

26. Zhang, N.; Wang, C.; Chen, J.; Hu, C.; Ma, J.; Deng, X.; Qiu, B.; Cai, L.; Xiong, Y.; Chai, Y., Metal substitution steering electron correlations in pyrochlore ruthenates for efficient acidic water oxidation. *ACS Nano* **15**, 8537–8548 (2021).

27. Xu, J.; Li, J.; Lian, Z.; Araujo, A.; Li, Y.; Wei, B.; Yu, Z.; Bondarchuk, O.; Amorim, I.; Tileli, V.; Li, B.; Liu, L., Atomic-step enriched ruthenium–iridium nanocrystals anchored homogeneously on MOF-derived support for efficient and stable oxygen evolution in acidic and neutral media. *ACS Catal.* **11**, 3402–3413 (2021).

28. Chen, L.; Li, Y.; Liang, X., Ar/H_2/O_2-controlled growth thermodynamics and kinetics to create zero-, one-, and two-dimensional ruthenium nanocrystals towards acidic overall water splitting. *Adv. Funct. Mater.* **31**, 2007344 (2021).

29. Xue, Y.; Fang, J.; Wang, X.; Xu, Z.; Zhang, Y.; Lv, Q.; Liu, M.; Zhu, W.; Zhuang, Z., Sulfate-functionalized $RuFeO_x$ as highly efficient oxygen evolution reaction electrocatalyst in acid. *Adv. Funct. Mater.* **31**, 2101405 (2021).

30. Zhang, H.; Wu, B.; Su, J.; Zhao, K.; Chen, L., MOF-derived zinc-doped ruthenium oxide hollow nanorods as highly active and stable electrocatalysts for oxygen evolution in acidic media. *ChemNanoMat* **7**, 117–121 (2021).

31. Yao, Q.; Huang, B.; Xu, Y.; Li, L.; Shao, Q.; Huang, X., A chemical etching strategy to improve and stabilize RuO_2-based nanoassemblies for acidic oxygen evolution. *Nano Energy* **84**, 105909 (2021).

32. Zhao, Z. L.; Wang, Q.; Huang, X.; Feng, Q.; Gu, S.; Zhang, Z.; Xu, H.; Zeng, L.; Gu, M.; Li, H., Boosting oxygen evolution reaction using defect-rich ultra-thin ruthenium oxide nanosheets in acidic media. *Energy Environ. Sci.* **13**, 5143–5151 (2020).

33. Kuznetsov, D. A.; Naeem, M. A.; Kumar, P. V.; Abdala, P. M.; Fedorov, A.; Muller, C. R., Tailoring lattice oxygen binding in ruthenium pyrochlores to enhance oxygen evolution activity. *J. Am. Chem. Soc.* **142**, 7883–7888 (2020).

34. Hao, S.; Liu, M.; Pan, J.; Liu, X.; Tan, X.; Xu, N.; He, Y.; Lei, L.; Zhang, X., Dopants fixation of ruthenium for boosting acidic oxygen evolution stability and activity. *Nat Commun.* **11**, 5368 (2020).

35. Cui, X.; Ren, P.; Ma, C.; Zhao, J.; Chen, R.; Chen, S.; Rajan, N. P.; Li, H.; Yu, L.; Tian, Z.; Deng, D., Robust interface Ru centers for high-performance acidic oxygen evolution. *Adv. Mater.* **32**, 1908126 (2020).

36. Chen, D.; Liu, T.; Wang, P.; Zhao, J.; Zhang, C.; Cheng, R.; Li, W.; Ji, P.; Pu, Z.; Mu, S., Ionothermal route to phase-pure RuB_2 catalysts for efficient oxygen evolution and water splitting in acidic media. *ACS Energy Lett.* **5**, 2909–2915 (2020).

37. Chen, S.; Huang, H.; Jiang, P.; Yang, K.; Diao, J.; Gong, S.; Liu, S.; Huang, M.; Wang, H.; Chen, Q., Mn-doped RuO_2

nanocrystals as highly active electrocatalysts for enhanced oxygen evolution in acidic media. *ACS Catal.* **10**, 1152–1160 (2020).

38. Xu, J.; Lian, Z.; Wei, B.; Li, Y.; Bondarchuk, O.; Zhang, N.; Yu, Z.; Araujo, A.; Amorim, I.; Wang, Z.; Li, B.; Liu, L., Strong electronic coupling between ultrafine iridium–ruthenium nanoclusters and conductive, acid-stable tellurium nanoparticle support for efficient and durable oxygen evolution in acidic and neutral media. *ACS Catal.* **10**, 3571–3579 (2020).

39. Wang, J.-Q.; Xi, C.; Wang, M.; Shang, L.; Mao, J.; Dong, C.-K.; Liu, H.; Kulinich, S. A.; Du, X.-W., Laser-generated grain boundaries in ruthenium nanoparticles for boosting oxygen evolution reaction. *ACS Catal.* **10**, 12575–12581 (2020).

40. Yao, Y.; Hu, S.; Chen, W.; Huang, Z.-Q.; Wei, W.; Yao, T.; Liu, R.; Zang, K.; Wang, X.; Wu, G.; Yuan, W.; Yuan, T.; Zhu, B.; Liu, W.; Li, Z.; He, D.; Xue, Z.; Wang, Y.; Zheng, X.; Dong, J.; Chang, C.-R.; Chen, Y.; Hong, X.; Luo, J.; Wei, S.; Li, W.-X.; Strasser, P.; Wu, Y.; Li, Y., Engineering the electronic structure of single atom Ru sites via compressive strain boosts acidic water oxidation electrocatalysis. *Nat. Catal.* **2**, 304–313 (2019).

41. Yao, Q.; Huang, B.; Zhang, N.; Sun, M.; Shao, Q.; Huang, X., Channel-rich RuCu nanosheets for pH-universal overall water splitting electrocatalysis. *Angew. Chem. Int. Ed.* **58**, 13983–13988 (2019).

42. Shan, J.; Ling, T.; Davey, K.; Zheng, Y.; Qiao, S.-Z., Transition-metal-doped RuIr bifunctional nanocrystals for overall water splitting in acidic environments. *Adv. Mater.* **31**, 1900510 (2019).

43. Ge, R.; Li, L.; Su, J.; Lin, Y.; Tian, Z.; Chen, L., Ultrafine defective RuO_2 electrocatayst integrated on carbon cloth for robust water oxidation in acidic media. *Adv. Energy Mater.* **9**, 1901313 (2019).

44. Laha, S.; Lee, Y.; Podjaski, F.; Weber, D.; Duppel, V.; Schoop, L. M.; Pielnhofer, F.; Scheurer, C.; Müller, K.; Starke, U.; Reuter, K.; Lotsch, B. V., Ruthenium oxide nanosheets for enhanced oxygen evolution catalysis in acidic medium. *Adv. Energy Mater.* **9**, 1803795 (2019).

45. Lin, Y.; Tian, Z.; Zhang, L.; Ma, J.; Jiang, Z.; Deibert, B. J.; Ge, R.; Chen, L., Chromium-ruthenium oxide solid solution electrocatalyst for highly efficient oxygen evolution reaction in acidic media. *Nat. Commun.* **10**, 162 (2019).

46. Miao, X.; Zhang, L.; Wu, L.; Hu, Z.; Shi, L.; Zhou, S., Quadruple perovskite ruthenate as a highly efficient catalyst for acidic water oxidation. *Nat. Commun.* **10**, 3809 (2019).

47. Retuerto, M.; Pascual, L.; Calle-Vallejo, F.; Ferrer, P.; Gianolio, D.; Pereira, A. G.; García, Á.; Torrero, J.; Fernández-Díaz, M. T.; Bencok, P.; Peña, M. A.; Fierro, J. L. G.; Rojas, S., Na-doped ruthenium perovskite electrocatalysts with improved oxygen evolution activity and durability in acidic media. *Nat. Commun.* **10**, 2041 (2019).

48. Wang, J.; Han, L.; Huang, B.; Shao, Q.; Xin, H. L.; Huang, X., Amorphization activated ruthenium-tellurium nanorods for efficient water splitting. *Nat. Commun.* **10**, 5692 (2019).

49. Cao, L.; Luo, Q.; Chen, J.; Wang, L.; Lin, Y.; Wang, H.; Liu, X.; Shen, X.; Zhang, W.; Liu, W.; Qi, Z.; Jiang, Z.; Yang, J.; Yao, T., Dynamic oxygen adsorption on single-atomic ruthenium catalyst with high performance for acidic oxygen evolution reaction. *Nat. Commun.* **10**, 4849 (2019).

50. Yu, J.; Li, G.; Liu, H.; Zhao, L.; Wang, A.; Liu, Z.; Li, H.; Liu, H.; Hu, Y.; Zhou, W., Ru–Ru_2P@NPC and NPC@RuO_2 synthesized via environment-friendly and solid-phase phosphating process by saccharomycetes as N/P sources and carbon template for overall water splitting in acid electrolyte. *Adv. Funct. Mater.* **29**, 1901154 (2019).

51. Su, J.; Ge, R.; Jiang, K.; Dong, Y.; Hao, F.; Tian, Z.; Chen, G.; Chen, L., Assembling ultrasmall copper-doped ruthenium oxide nanocrystals into hollow porous polyhedra: Highly robust electrocatalysts for oxygen evolution in acidic media. *Adv. Mater.* **30**, 1801351 (2018).

52. Gloag, L.; Benedetti, T. M.; Cheong, S.; Marjo, C. E.; Gooding, J. J.; Tilley, R. D., Cubic-core hexagonal-branch mechanism to synthesize bimetallic branched and faceted Pd–Ru nanoparticles for oxygen evolution reaction electrocatalysis. *J. Am. Chem. Soc.* **140**, 12760–12764 (2018).

53. Gloag, L.; Benedetti, T. M.; Cheong, S.; Li, Y.; Chan, X.-H.; Lacroix, L.-M.; Chang, S. L. Y.; Arenal, R.; Florea, I.; Barron, H.; Barnard, A. S.; Henning, A. M.; Zhao, C.; Schuhmann, W.; Gooding, J. J.; Tilley, R. D., Three-dimensional branched and faceted gold–ruthenium nanoparticles: Using nanostructure to improve stability in oxygen evolution electrocatalysis. *Angew. Chem. Int. Ed.* **57**, 10241–10245 (2018).

54. Shan, J.; Guo, C.; Zhu, Y.; Chen, S.; Song, L.; Jaroniec, M.; Zheng, Y.; Qiao, S.-Z., Charge-redistribution-enhanced nanocrystalline Ru@IrO_x electrocatalysts for oxygen evolution in acidic media. *Chem* **5**, 445–459 (2019).

55. Kim, J.; Shih, P.-C.; Qin, Y.; Al-Bardan, Z.; Sun, C.-J.; Yang, H., A porous pyrochlore $Y_2[Ru_{1.6}Y_{0.4}]O_{7-\delta}$ electrocatalyst for enhanced performance towards the oxygen evolution reaction in acidic media. *Angew. Chem. Int. Ed.* **57**, 13877–13881 (2018).

56. Cherevko, S.; Geiger, S.; Kasian, O.; Kulyk, N.; Grote, J.-P.; Savan, A.; Shrestha, B. R.; Merzlikin, S.; Breitbach, B.; Ludwig, A.; Mayrhofer, K. J. J. Oxygen and hydrogen evolution reactions on Ru, RuO_2, Ir, and IrO_2 thin film electrodes in acidic and alkaline electrolytes: A comparative study on activity and stability. *Catal. Today* **262**, 170-180 (2016).

57 Hubert, M. A.; Patel, A. M.; Gallo, A.; Liu, Y.; Valle, E.; Ben-Naim, M.; Sanchez, J.; Sokaras, D.; Sinclair, R.; Nørskov, J. K.; King, L. A.; Bajdich, M.; Jaramillo, T. F. Acidic oxygen evolution reaction activity–stability relationships in Ru-based pyrochlores. *ACS Catal.* **10**, 12182–12196 (2020).
